# Fungal hyphae develop where titanomagnetite inclusions reach the surface of basalt grains

**DOI:** 10.1038/s41598-021-04157-z

**Published:** 2022-03-01

**Authors:** Rebecca A. Lybrand, Odeta Qafoku, Mark E. Bowden, Michael F. Hochella, Libor Kovarik, Daniel E. Perea, Nikolla P. Qafoku, Paul A. Schroeder, Mark G. Wirth, Dragos G. Zaharescu

**Affiliations:** 1 Department of Crop and Soil Science, Oregon State University, 109 Crop Science Building, 3050 SW Campus Way, Corvallis, OR 97331 USA; 2 Pacific Northwest National Laboratory, 902 Batelle Blvd, Richland, WA 99354 USA; 3 Department of Geosciences, Virginia Tech, Blacksburg, VA 24061 USA; 4 Department of Geology, University of Georgia, 210 Field Street, Athens, GA 30602 USA; 5 Department of Natural and Applied Sciences, University of Wisconsin, 2420 Nicolet Dr, Green Bay, WI 54311 USA; 6 Department of Land, Air & Water Resources, University of California, Davis, One Shields Avenue, Davis, CA 95616 USA

**Keywords:** Biogeochemistry, Biogeochemistry, Solid Earth sciences

## Abstract

Nutrient foraging by fungi weathers rocks by mechanical and biochemical processes. Distinguishing fungal-driven transformation from abiotic mechanisms in soil remains a challenge due to complexities within natural field environments. We examined the role of fungal hyphae in the incipient weathering of granulated basalt from a three-year field experiment in a mixed hardwood-pine forest (S. Carolina) to identify alteration at the nanometer to micron scales based on microscopy-tomography analyses. Investigations of fungal-grain contacts revealed (i) a hypha-biofilm-basaltic glass interface coinciding with titanomagnetite inclusions exposed on the grain surface and embedded in the glass matrix and (ii) native dendritic and subhedral titanomagnetite inclusions in the upper 1–2 µm of the grain surface that spanned the length of the fungal-grain interface. We provide evidence of submicron basaltic glass dissolution occurring at a fungal-grain contact in a soil field setting. An example of how fungal-mediated weathering can be distinguished from abiotic mechanisms in the field was demonstrated by observing hyphal selective occupation and hydrolysis of glass-titanomagnetite surfaces. We hypothesize that the fungi were drawn to basaltic glass-titanomagnetite boundaries given that titanomagnetite exposed on or very near grain surfaces represents a source of iron to microbes. Furthermore, glass is energetically favorable to weathering in the presence of titanomagnetite. Our observations demonstrate that fungi interact with and transform basaltic substrates over a three-year time scale in field environments, which is central to understanding the rates and pathways of biogeochemical reactions related to nuclear waste disposal, geologic carbon storage, nutrient cycling, cultural artifact preservation, and soil-formation processes.

## Introduction

The weathering of basalts and natural glasses has wide-ranging implications for our society and beyond including the supply of life-supporting nutrients from rocks to the environment^1^. Over geologic timescales, the global consumption of atmospheric carbon dioxide by basaltic weathering sequesters carbon into stable carbonate rock, which is twice as efficient as weathering carbonate rocks themselves^2,3^. The coevolution of the geosphere-biosphere on early Earth was also linked to the weathering of basalt and the formation of clay minerals^4,5^. With respect to the study of planetary systems, the degree of basaltic alteration observed on Mars contributes to our understanding of astrobiology and the evolution of extraterrestrial landscapes^6–8^. The pathways and mechanisms that control the dissolution of synthetic glasses produced for the storage of radionuclides in geologic repositories has also been well-studied^9,10^. Thus, assessing the biotic and abiotic drivers of basalt and glass weathering in natural Earth systems, including soils, remains of paramount importance for recognizing and predicting long-term ecosystem response to changes in global climate^11,12^, identifying lithologic controls on global carbon cycling^2,13^, or detecting mineralogical biosignatures that may provide evidence for past, present, and future life on other planets^14–16^.

Emphasis has been placed on the importance of micro- to nanoscale investigations for disentangling the intricacies of microbially-mediated or abiotically-driven weathering mechanisms^17,18^, particularly in complex ecosystems where laboratory studies do not reproduce the extent of weathering that occurs in soils^19^. Microorganisms catalyze mineral weathering and silicate glass alteration to varying extents^20–23^, yet it remains unclear to what degree biological weathering processes transform volcanic glass and basaltic minerals^24,25^. Laboratory studies examining the relative influence of abiotic and biotic constituents on basalt dissolution report a number of realizations that include: (1) greater rates of basalt dissolution in the presence of bacteria where the degree of weathering also varied with the bulk elemental compositions of the volcanic glasses^26,27^; (2) the enhanced release of Zr, Sc, Mn, Fe, Ti, Si/Al, and Si/Fe from basalt in the presence of organic acids, citrate, and dissolved organic matter extracts from Ponderosa Pine forest soil organic horizons^28,29^; and (3) the important role of biofilms in producing contrasting secondary weathering products under abiotic versus biotic conditions^24^. Microbially-mediated weathering processes have been further explored by examining the formation of bioalteration textures in basaltic glasses from ocean basins^30,31^. In fact, the morphological features of bioalteration textures were used as evidence for microbial activity in basalt transformation in the Hawaii Scientific Drilling Program^32–34^. Incipient weathering experiments conducted under abiotic and biotic conditions showed that microbes enhanced basaltic weathering (i.e., hydrolyzed, dissolved and precipitated major matrix elements) and stimulated the precipitation of secondary Fe and Mn minerals while decreasing the mobilization of redox-sensitive Fe and Mn to pore water (e.g., putative biogenic manganese oxides^27^). However, biological weathering remains understudied in soils and in rocks exposed to field conditions (e.g.,^35^), particularly at nano- to micron scales where high-resolution microscopy and tomography capabilities provide the opportunity to investigate biotic processes in great detail^36,37^.

Fungal weathering influences mineral dissolution and transformation processes in laboratory settings^21,38–40^ and in field settings^41–44^. Additional evidence from laboratory studies indicate that mycorrhizal fungi act as bio-sensors that selectively allocate carbon to favor growth towards different particle size fractions (53–90 µm; 500–1000 µm) and specific minerals, e.g., apatite (e.g., source of P) and biotite (e.g., source of K and Fe^21,45^). Chemical energy transfer from plants to mycorrhizal fungi enhances rates of calcium dissolution from basalt as shown in a set of ^14^CO_2_-tracer experiments performed at near-current (450 ppm) and past high atmospheric CO_2_ conditions in environmental growth chambers (1500 ppm^46^). To address the complexities of fungal distribution under natural soil conditions, in-soil mesh bags have served as a tool to assess ectomycorrhizal contributions to weathering, carbon turnover, and fungal response to organic and inorganic nutrient sources in forest soils^47–49^. Lybrand et al. (2019) deployed granulated rock substrates (250–53 µm) in granitic soils to assess incipient weathering along a climate gradient spanning from semiarid to humid ecosystems^41^. Three substrate types were set up for the study including (1) quartz sand as a nutrient-poor, in-situ control to assess background levels of fungal foraging; (2) granite extracted from a granitic rock quarry in southern Arizona to provide a fresh supply of coarse-grained minerals^27,50^; and (3) basalt, given its importance as a silicate rock for global carbon and nutrient cycling^46^. Moreover, basaltic minerals represented an easily accessible, fine-grained source of nutrients in granitic terrain. Results provided evidence for fungal-driven biomechanical weathering of the granite and basalt in all three ecosystems after one year of field burial^41^, and highlighted the need for more in-depth assessments of biotic-mineral interfaces, particularly on basaltic surfaces inhabited by fungi and containing mineral coatings presumably from microbial activity^51^.

Our objective for this study was to interrogate microbe-mineral interactions in natural environments to offer new perspectives on the alteration of basaltic materials in a forest soil system. We performed a micro- to nanoscale investigation of fungal-grain contacts on basaltic samples collected from an incipient weathering experiment in a mixed hardwood-pine forest. Basaltic granulated substrates were sealed into nylon mesh bags, deployed in surface mineral soils, and exposed to soil microbes for three years. High resolution microscopy was employed to characterize the morphological nature of the microbe-grain interfaces in the retrieved granulated basaltic substrate and to map elemental distribution. A subset of select fungal-grain interfacial boundaries were examined with Focused Ion Beam/Scanning Electron Microscopy (FIB/SEM) and Transmission Electron Microscopy (TEM) to test the hypothesis that Fe-rich minerals near fungal hyphae formed from fungal-mediated biomineralization processes. We present evidence for incipient weathering along fungal-grain contacts and findings from the assessment of inclusions in the basaltic glass matrix. We also investigated three-dimensional elemental distribution within a basaltic glass matrix and across an interfacial boundary with titanomagnetite crystals at near atomic scale resolution provided by Atom Probe Tomography (APT).

## Results

### Assessment of basaltic substrate following exposure to field weathering conditions

**Figure 1:**
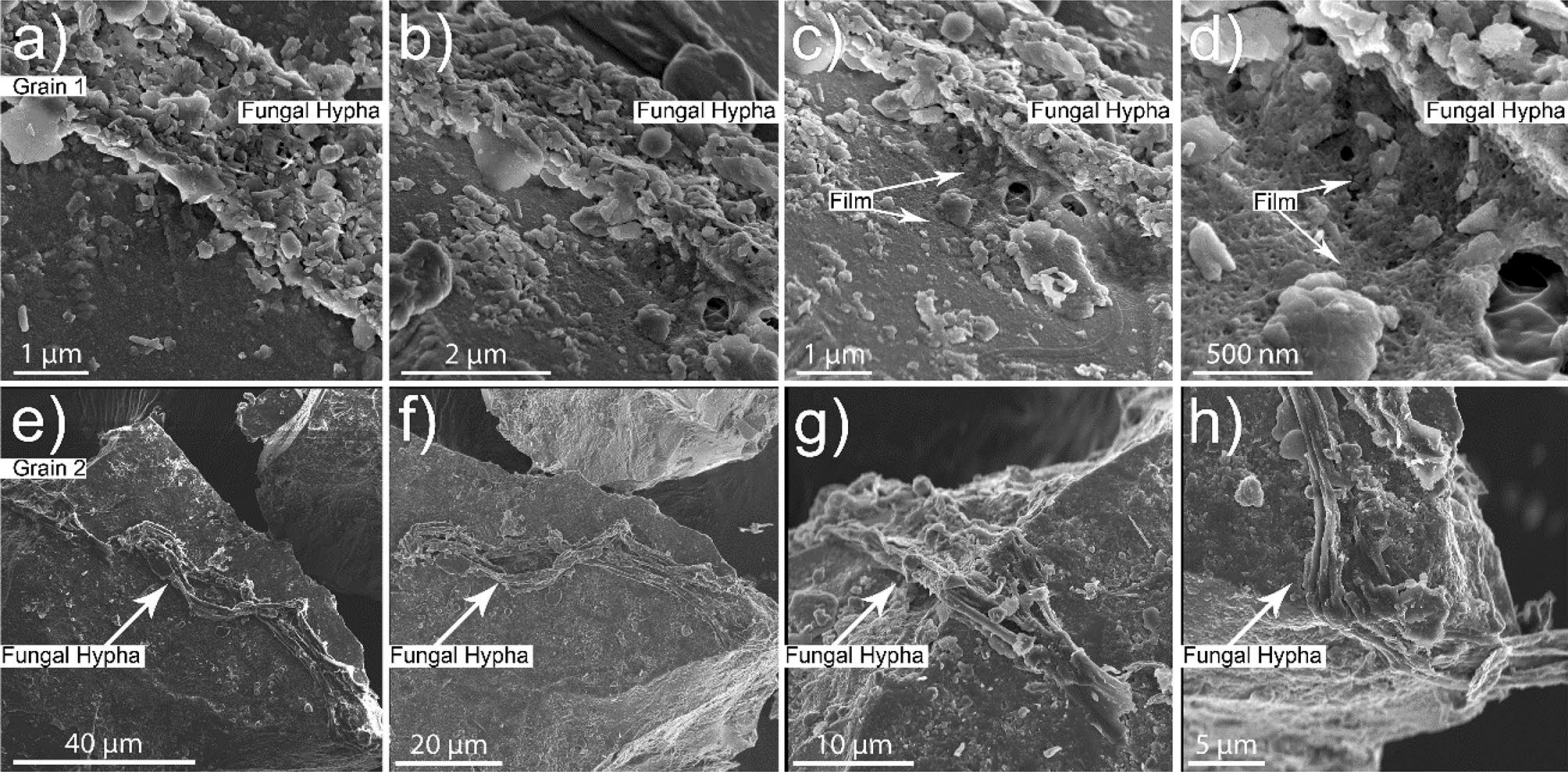
Micrographs that show fungal growth on (**a**–**d**) Grain 1 and (**e**–**h**) Grain 2 following deployment in a mixed hardwood forest for 3 years (Calhoun Experimental Forest, South Carolina). (**c**) and (**d**) are higher resolution micrographs of (**b**). (**g**) and (**h**) are higher resolution micrographs of (**e**). The higher magnifications in (**c**) and (**d**) display the fungal-grain contact and the presence of an interwoven mat or a sticky, organic film enclosing the sides of the fungal hypha. The higher magnifications for Grain 2 in (**g**) and (**h**) show close interactions of a fungal hypha and the edge of a basaltic grain.

Basaltic mesh bag samples showed fungal colonization and evidence for fungal-grain interactions following deployment at a mixed hardwood-pine forest site in South Carolina (Figs. 1, S1). High-resolution microscopy surveys of five grains revealed strands of fungal hyphae adhering to grain surfaces and growing along grain edges (Figs. 1, S1). The grains comprised a basaltic glass matrix with Fe oxide or Fe-rich inclusions as detected with Energy Dispersive X-ray Spectroscopy (EDX) point analyses across each grain surface where fungal-grain contacts were observed (Table S1). The fungal structures themselves appeared covered in particulate materials and submicron sized mineral particles (Fig. 1a–h). In some instances, the fungal hypha was enclosed in an organic film or interwoven mat on the grain surface that appeared to be a biofilm based on comparisons to morphological features from prior work (Fig. 1a–d^52^).

**Figure 2:**
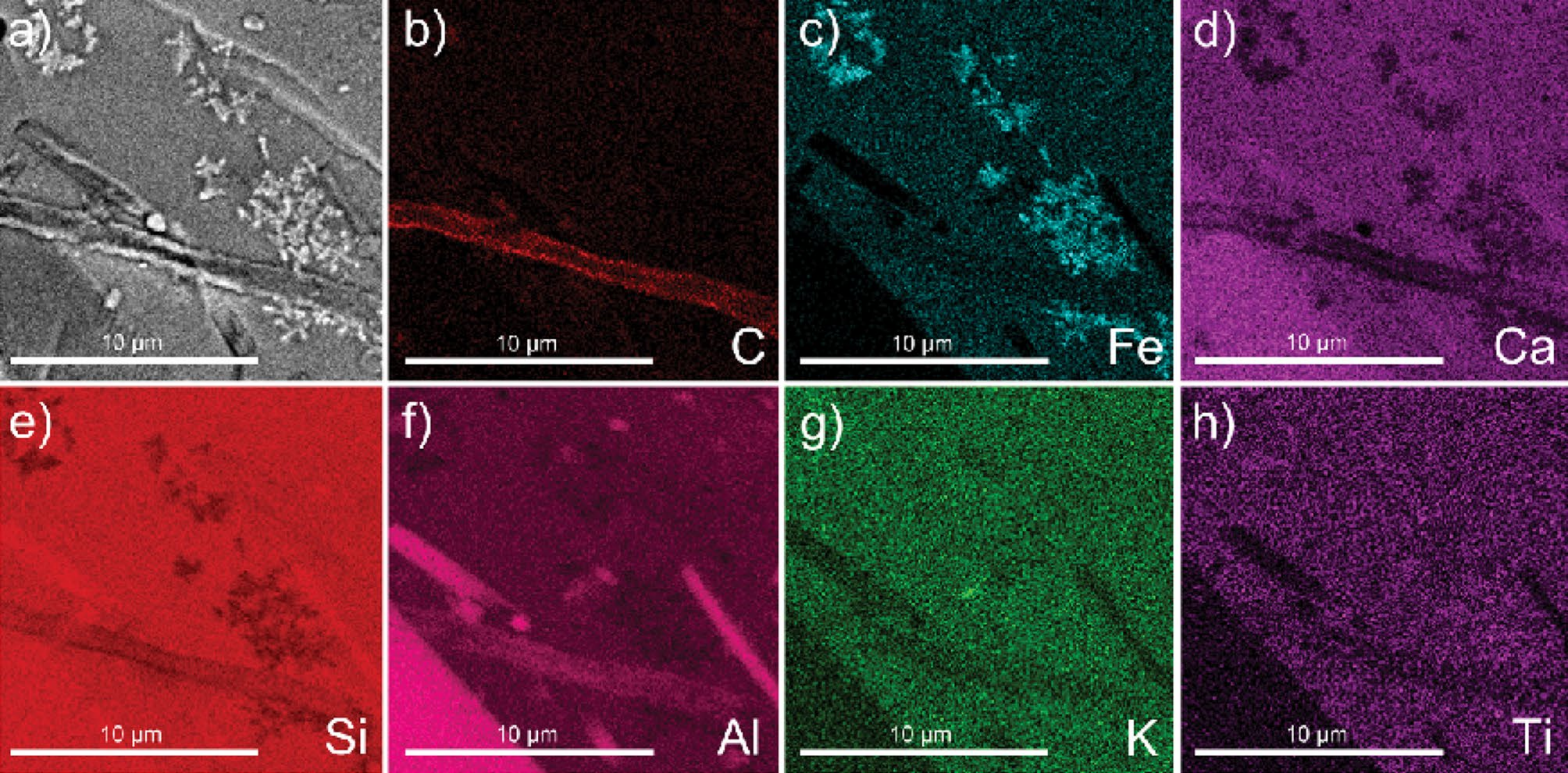
Micrograph and elemental maps for Grain 1 showing surface morphology of the grain adjacent to a fungal hypha after exposure to forest soil conditions for three years. We note the presence of Fe-rich particles oriented in dendritic-like habits that show high Z-contrast that were later identified as titanomagnetite, and possible evidence for additional feldspar mineral inclusions. The element presented in each elemental map is labelled in the bottom right corner of each subfigure. The brighter colors in the elemental maps indicate a higher abundance of that element compared to darker regions in the same maps.

**Figure 3:**
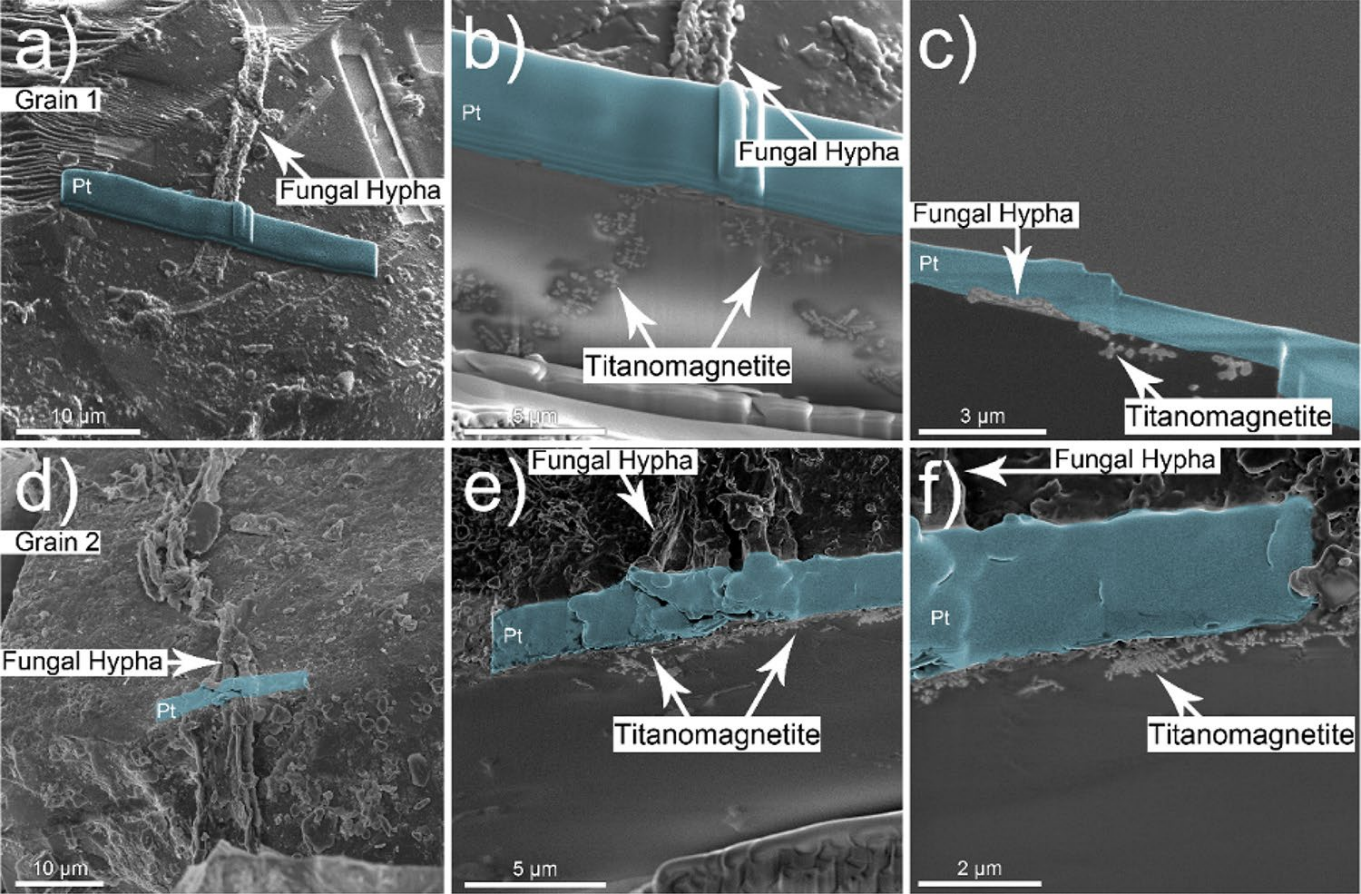
A series of FIB/SEM cross-sections at the two fungal-grain interfaces identified on Grain 1 (top row) and Grain 2 (bottom row). (**a**–**c**) The cross-sections reveal evidence of submicron size titanomagnetite particles in the near-surface of the grain that display similar morphological traits as titanomagnetite particles found on the surface of Grain 1. (**d**–**f**) Submicron size titanomagnetite particles appear along the fungal-grain contact for Grain 2 that extend laterally and vertically along and underneath the interface. Pt deposition/protection layer in false-colored blue.

We focused our microscopy-tomography investigation on fungal-grain interactions in two grains from our initial survey, referenced herein as Grain 1 and Grain 2 (Fig. 1; see “Methods”), both of which were composed of the basaltic glass matrix with inclusions of minerals. We selected a fungal-grain interface on Grain 1 that was embedded in an interwoven mat or sticky biofilm coating (Fig. 1d) and occurred in association with possible feldspar inclusions (Fig. 2). SEM/EDX elemental maps for Grain 1 revealed sub-micron-sized Fe- and Ti–rich crystals adjacent to the fungal-grain attachment that were initially hypothesized to be formed from fungal-driven biomineralization processes (Figs. 2; Fig. S2–S5; Table S1). To address whether the Fe-Ti minerals coincided with other sites of fungal activity on basaltic glass, we selected a second grain (i.e., Grain 2) that was also colonized by fungal hyphae (Fig. 1e–h). The basaltic glass matrix of Grain 2 presented similar elemental compositions to Grain 1 (Table S1); however, we did not observe Fe-Ti crystals exposed on the surface as with Grain 1. Surprisingly, we incidentally discovered the same Fe-Ti crystals embedded in the matrix of Grain 2 beneath the fungal-grain contact despite their absence on the grain surface (Fig. 3). We selected both fungal-mineral interfaces on Grains 1 and 2 to investigate further using a SEM/FIB and TEM approach.

### Examining fungal-grain interfaces in basaltic substrate

**Figure 4:**
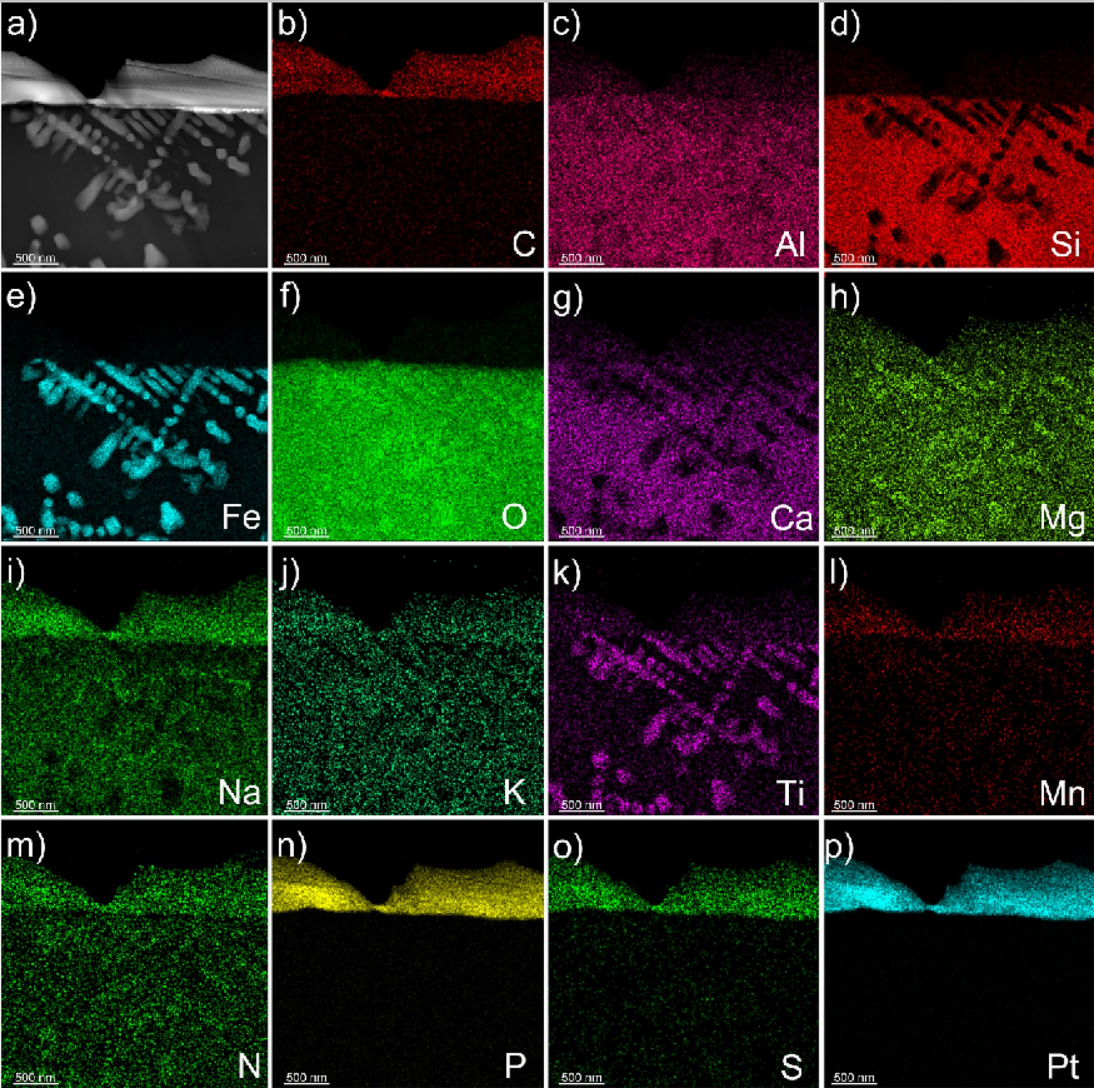
Micrograph and elemental maps generated by TEM analyses for Grain 1 show the distribution of nanosized titanomagnetite in the near-surface of Grain 1. A protective Pt layer was added during the sample preparation process to cap and protect the location of the vertical cross-section milled into the grain surface. The length of the Pt deposit is displayed in light blue as part of (**p**). Grain 1 is representative of a basaltic glass matrix with embedded mineral inclusions, such as titanomagnetite. The elemental maps were produced approximately 5–7 µm from the fungal-grain contact zone where the precise location of this lamella extraction occurred in the upper right corner of Fig. 3a. The element presented in each elemental map is labelled in the bottom right corner of each subfigure. The brighter colors in the elemental maps indicate a higher abundance of that element compared to darker regions in the same maps.

**Figure 5:**
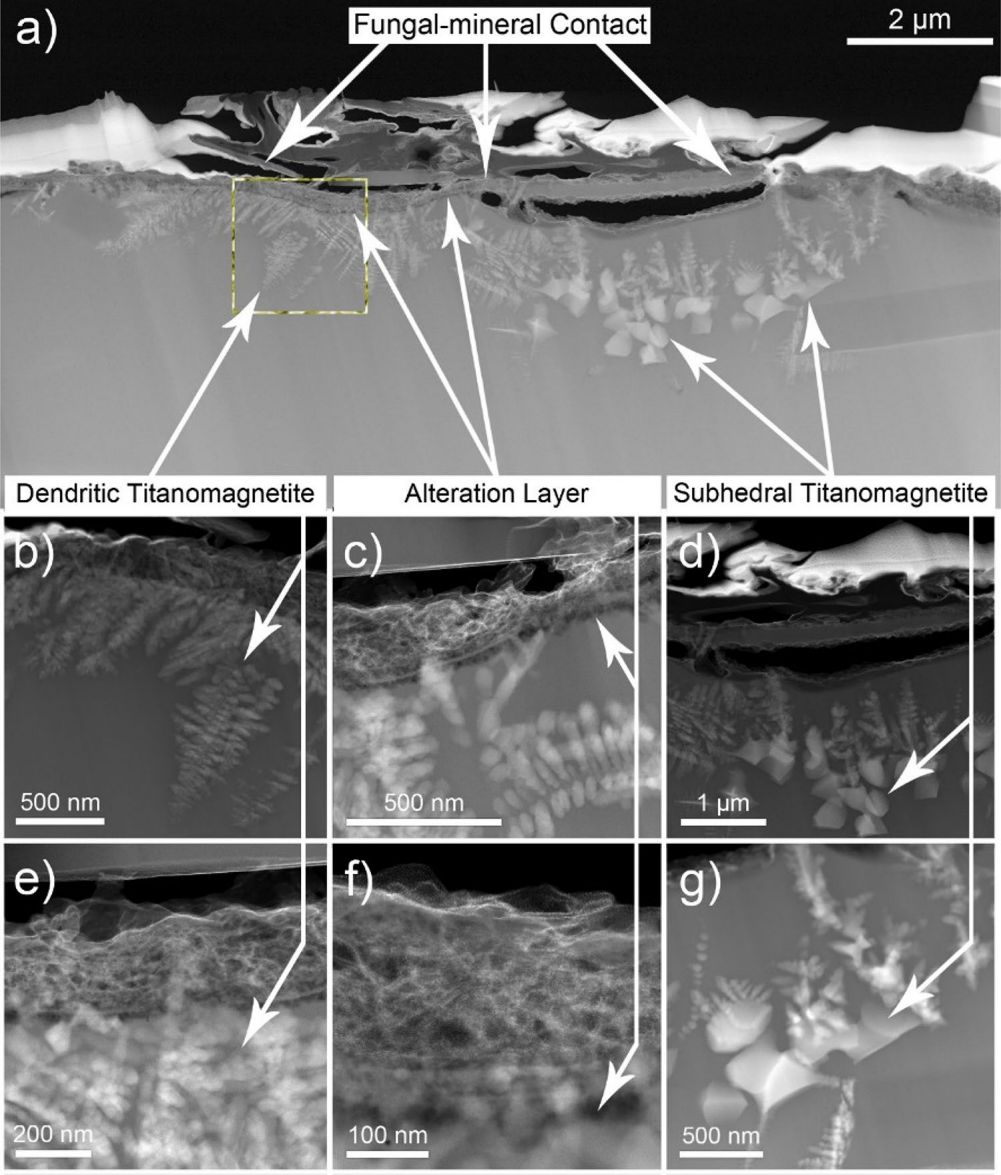
SEM/FIB vertical cross-section of Grain 2 showing (**a**) a concave shape along the grain surface exposed to direct contact with the fungal hypha. The dashed yellow line highlights the region of the interface that corresponds to the elemental maps presented in Fig. 6b–g). Micrographs show magnifications of the fungal-grain contact including inclusions formed as (**b**, **e**) dendritic and (**d**, **g**) subhedral titanomagnetite crystals that were identified along the surface of the basaltic grain at and near the fungal-grain interface. (**c**, **f**) A thin, dark alteration layer stretched across the grain surface suggesting evidence for incipient weathering of the basaltic grain.

**Figure 6:**
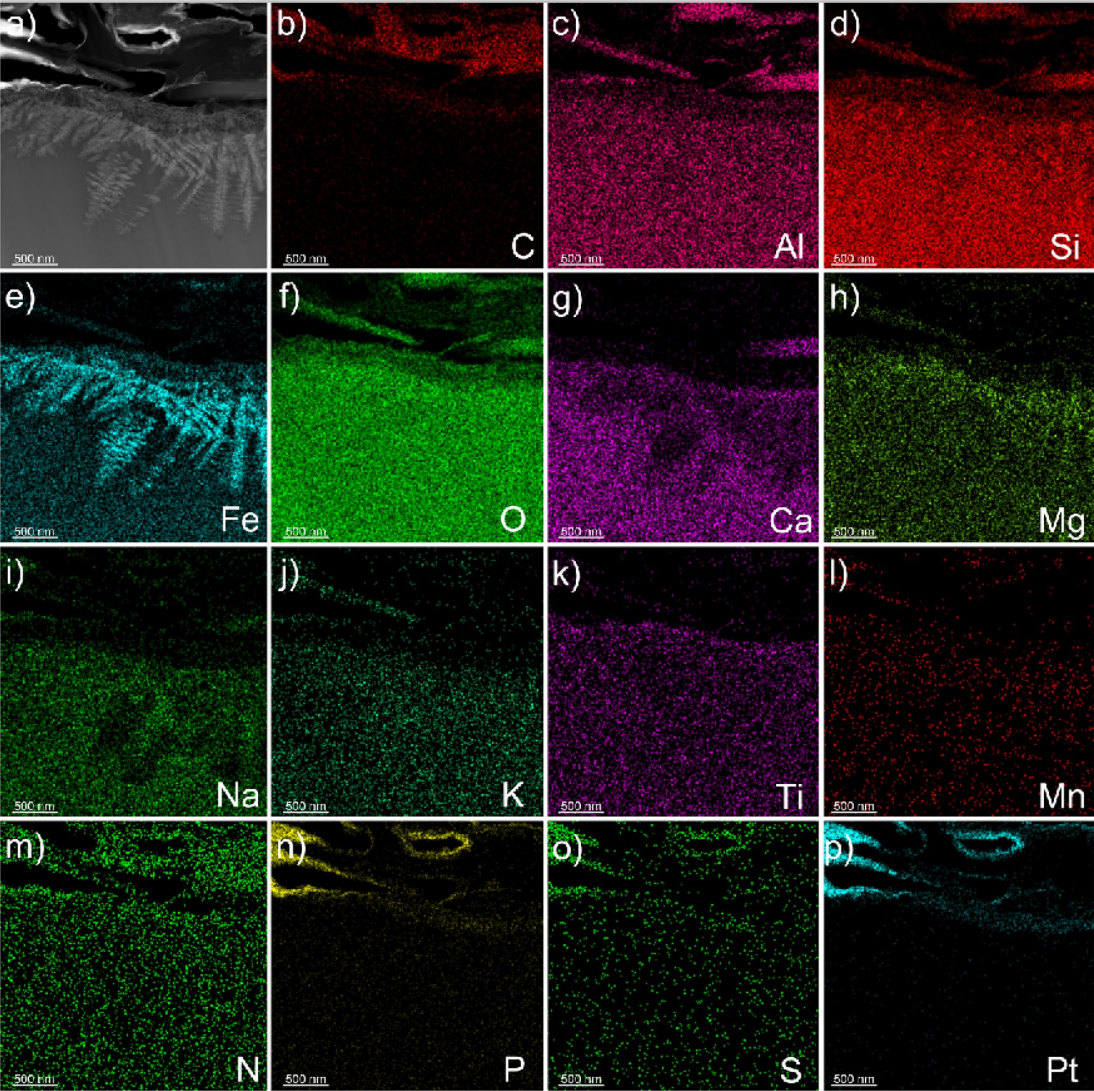
(**a**) Electron micrograph and (**b**–**p**) elemental maps generated by TEM for a cross section on Grain 2 that show nanosized dendritic titanomagnetite inclusions extending laterally along the fungal-grain contact. The element presented in each elemental map is labelled in the bottom right corner of each subfigure. The brighter colors in the elemental maps indicate a higher abundance of that element compared to darker regions in the same maps. The top of the lamella is protected by a Pt layer. The length of the Pt deposit is displayed in light blue as part of (**p**).

Vertical cross sections were milled into Grains 1 and 2 at each fungal-grain interface using FIB/SEM (Fig. 3), which revealed associations between fungi and the Fe-Ti crystals observed in the elemental maps for the surface of Grain 1 (Fig. 2) and the occurrence of crystals in the grains themselves (Grain 1, 2; Fig. 3). The Fe-Ti crystals in Grains 1 and 2 were identified as dendritic titanomagnetite and larger subhedral single crystals of titanomagnetite based on electron diffraction patterns collected during TEM analysis (Fig. S8). Elemental maps generated by TEM analyses displayed the distribution and morphology of the titanomagnetite crystals in Grain 1 (Figs. 4, S2–S5) and Grain 2 (Figs. 5, 6, S6, S7).

The titanomagnetite inclusions detected at the fungal-grain interface in Grain 1 appeared as dendrites, clusters, or chains of titanomagnetite crystals in straight, needle-like orientations^53^ (Figs. 3, 4, S4). We used Selected Area Electron Diffraction (SAED) analyses to confirm that the crystals were titanomagnetite given that the measured lattice spacings and angular relationship of the diffracted intensities are fully consistent with the diffraction patterns and the isometric lattice parameter of titanomagnetite (*a* = 8.4 Å). An example of self-consistently indexed diffraction patterns from the [103] zone axis of the titanomagnetite crystal is shown in Fig. S8. Titanomagnetite crystals were either exposed on the grain surface (Fig. 2) or extended ~ 5 µm into the grain where the crystals were perpendicular to the fungal-grain interface (Fig. S5b). The titanomagnetite contained elevated levels of Ti relative to the glass matrix (Table S1).

The fungal-grain interface for Grain 2 presented no evidence for titanomagnetite crystals exposed on the grain surface as observed for Grain 1 (Fig. 2e–h). Interestingly, analyses of the vertical cross sections of the lamella for Grain 2 confirmed the presence of titanomagnetite in at least two morphological forms (Figs. 5, 6). Dendritic titanomagnetite was the most visibly abundant form, where the crystals extended laterally near the grain surface at ~ 2 µm in depth and coincided with the fungal-grain interface (Figs. 5, S6, S7). Larger forms of titanomagnetite crystals with a subhedral morphology occurred further from the fungal contact at ~ 3–5 µm into the grain (Fig. 5a,d,g). The vertical cross section of Grain 2 also revealed additional ~ 1 µm sized titanomagnetite inclusions at ~ 10 µm depth that were also consistent with titanomagnetite (based on morphology and elemental maps; Fig. S7a). Similar formations have been observed in prior electron microprobe characterization of the unreacted basaltic substrate used herein^27,50^, and through electron microscopy investigations of complexly mixed magnetic mineral assemblages from deep-sea surface sediment samples^53^.

As for Grain 1, we identified the dendritic inclusions as titanomagnetite (SAED diffraction analyses in combination with EDX measurements). An example of a self-consistently indexed diffraction pattern from [110] zone axis is shown in Fig. S8. The angular relationship of the diffracted intensities is consistent with Grain 1 and showed agreement with an indexed titanomagnetite lattice parameter of *a* = 8.4 Å. SAED patterns taken from the center of the titanomagnetite crystals are shown in Fig. S8c and g. Similar to the titanomagnetite inclusions in Grain 1, titanomagnetite crystals in Grain 2 are enriched in Ti relative to the surrounding basaltic glass matrix (Table S1).

**Figure 7:**
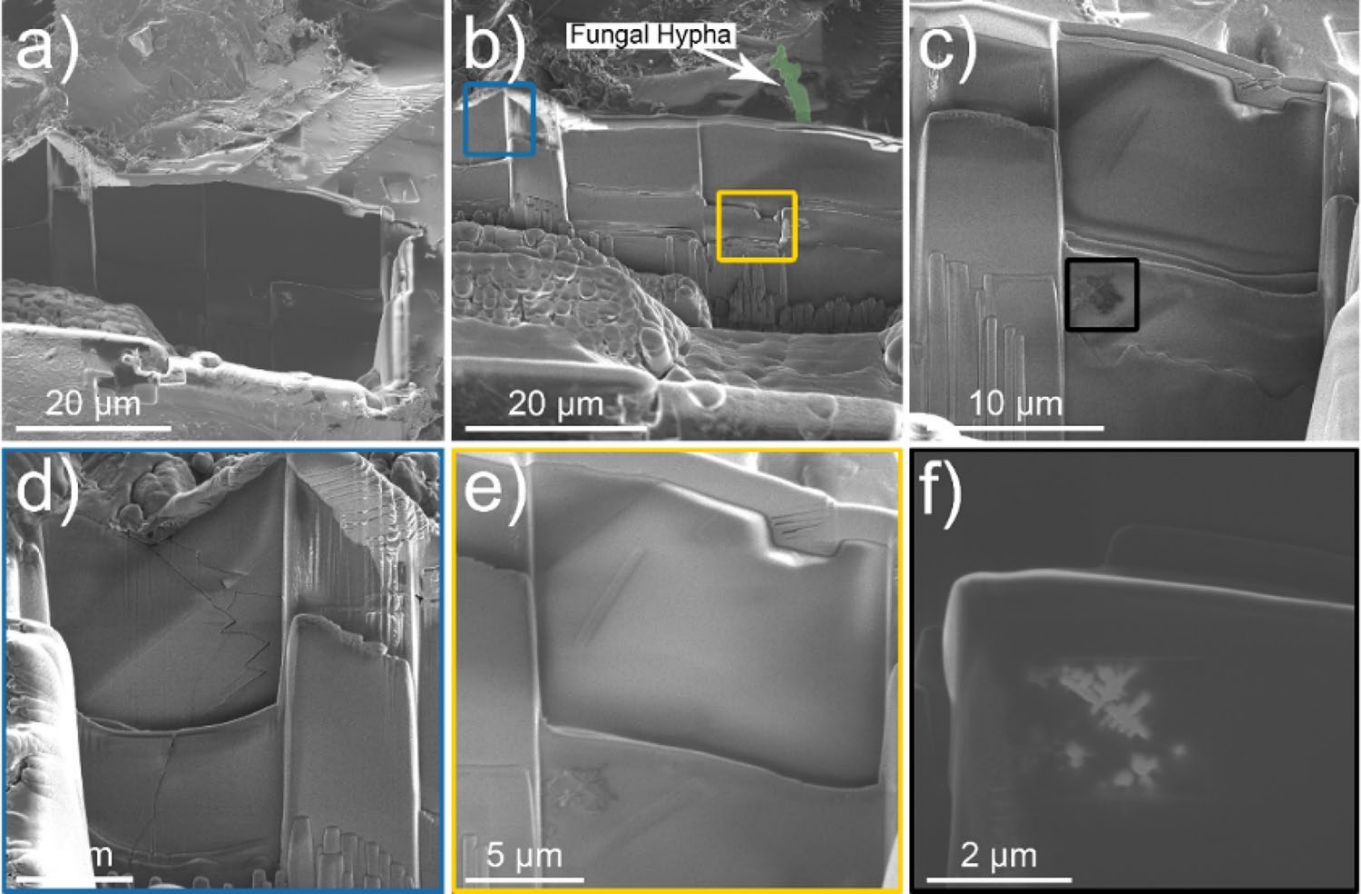
(**a**) Extended trench showing no evidence for grain alteration or the presence of titanomagnetite crystals at the grain surface. (**b**) The white arrow marks the location of a fungal hypha on the grain surface (hypha denoted by false-colored green) and (**c**) the location of a magnetite inclusion at depth. (**d**) The blue box provides an example of the grain surface with no near surface magnetite crystals or fungal-grain contacts. The magnetite inclusion highlighted in (**c**) occurred at ~ 10 µm into the grain as shown with magnified images contained in (**e**) yellow and (**f**) black boxes.

The cross-sectional view of Grain 2 presents evidence for a concavely shaped contact at the fungal-grain interface (Fig. 5a) and an alteration layer extending across the surface of the basaltic grain (Fig. 5a,c,f). The concave shape and associated fungal contact with the grain was observed before and during the FIB process. The fungal-grain interface containing the concave feature was preserved through FIB milling, thinning, and sample preparation where the fungal material and the majority of the protective platinum (Pt) cap above the interface was preserved as evidenced in Fig. S9. Such depressions in the surface were not observed at the fungal-grain interface in Grain 1 (Figs. 3, S3, S4) or along on extended trench opened on Grain 1 to investigate the nature of grain surfaces uninhabited by fungi (Fig. 7). The trench was opened on areas of the grain where we observed no evidence for direct fungal-grain activity and no titanomagnetite crystals exposed at or near the grain surface (Fig. 7).

### Three-dimensional elemental distribution along basaltic glass-titanomagnetite boundaries

**Figure 8:**
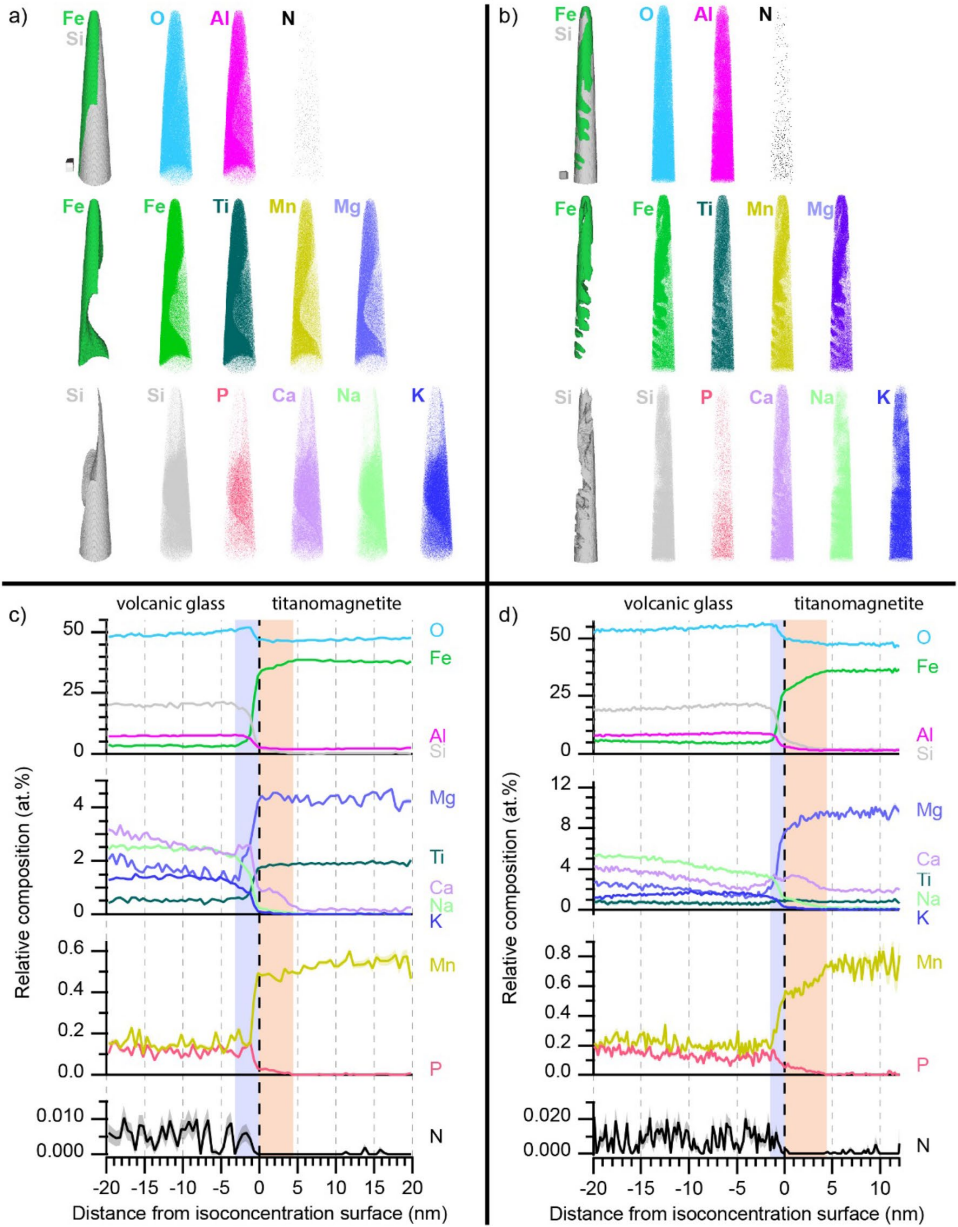
APT 3D composition point cloud maps and 1D composition profiles (proximity histograms) for Grain 1 (**a**, **c**) and Grain 2 (**b**, **d**) showing homogenous elemental distribution along the glass-titanomagnetite interface including a subtle enrichment of Ca. Additional APT figures, videos, and tables are presented in Figs. S10–S12 and Table S3. The proximity histograms for (**c**) Grain 1 and (**d**) Grain 2 are set at the interface (defined by 15% and 16% Fe isoconcentration surfaces (x = 0 nm), respectively. Compositions within the Si-rich phase are to the left of zero, while compositions within the Fe-rich phase are to the right of zero. The error in each composition profile, as determined by counting error, is indicated by an overlaid shaded error band of a corresponding muted color, which is most noticeable for the N profile containing a grey error band. The scale cubes shown in (**a**) and (**b**) correspond to 20 × 20 × 20 nm^3^.

Using Atomic Probe Tomography (APT) to probe the distribution of elements in the glass matrix and titanomagnetite phases in Grain 1 and Grain 2 allows for a specific focus on examining the basaltic glass-titanomagnetite near and beneath the exposed fungal-grain interface (Fig. 8). We examined the atomic-scale distribution of elements within separate volumes as intersecting the interfacial boundary separating the two phases (Figs. 8, S10–S12) as well as entirely within the basaltic glass matrix adjacent to the titanomagnetite crystals (Figs. S10, S11) for both Grain 1 and Grain 2. Our interrogations focused on the nanosized titanomagnetite inclusions near the fungal-grain interface in Grain 1 (Fig. 8a,c) and those directly beneath the fungal contact in Grain 2 (Fig. 8b,d). When comparing three-dimensional (3-D) elemental composition point cloud maps and mass spectra from APT analyses along the interfacial boundaries, we found that the titanomagnetite crystals were enriched in Ti, and to a lesser extent, Mg and Mn in both Grain 1 and Grain 2 (Fig. 8, Table S3). The elements in the titanomagnetite were homogenously distributed throughout the inclusions in both grains (Fig. 8a,b). Conversely, the basaltic glass matrix comprised a greater abundance of heterogeneously distributed nutrients, such as Ca, K, Na, and P, among others, and traces of N (Fig. 8, Table S3).

Although the 3-D composition point cloud maps in Fig. 8a and b qualitatively show heterogeneity of the various elements partitioned between the basaltic glass and titanomagnetite phases, a proximity histogram (proxygram)^54^ provided quantitative 1-D composition profiles which follow the morphologically complex interface contours defined by an isoconcentration surface (Fig. 8c,d). The 1-D proxygram composition profiles show Fe, Mn, Ti, and Si all to exhibit relatively sharp, abrupt (approximately 2–3 nm) boundaries at the interfacial transition as highlighted within the vertical muted purple color bands of Fig. 8c,d. The orange and purple bands serve as a visual guide to delineate the spatial extent of the interface which span approximately the distance between the low and high steady-state Fe composition within the respective glass and titanomagnetite phases (Fig. 8c,d). The boundaries between phases were less abrupt for P (> 2 nm) and even more so for Ca (> 7 nm) as highlighted by the vertical muted orange colored bands (Fig. 8c,d) showing a monotonic decreasing composition of both Ca and P extending into the interface. However, Ca also exhibits a slight yet noticeable enrichment evidenced by a compositional hump within the interface boundary which is bounded by the muted orange colored bands for both grains. 3-D animations created across the basaltic glass-titanomagnetite phases also demonstrate the complex morphology of the heterointerface between the basaltic glass matrix and the titanomagnetite phases (Fig. S10).

## Discussion

### Nature of basaltic glass-titanomagnetite interfacial boundary

Titanomagnetite with dendritic and subhedral habits were identified within the basaltic glass matrices of Grains 1 and 2 (Figs. 2, 3, 4, 5, 6). We conclude that the subhedral and dendritic forms were already present in the grains as inclusions, as we found no microscale evidence to signify secondary bio-mineralization, i.e., grain stress, grain fractures, or dissolution fronts around the titanomagnetite crystals (Figs. 4, 5, 6^45^). Indeed, the morphological structures of the titanomagnetite inclusions in our work align well with established morphologies of magnetite reported previously that match abiotic formation and growth of abiotic iron oxide crystals in basaltic rocks (Figs. 4, 5, 6^55,56^) compared to chains of octahedral biomagnetite or magnetofossils formed during biotic processes^53,57^. Furthermore, based on magnetic and electron microscopy analyses of complex magnetite mixtures extracted from deep-sea surface sediments, prior research concluded that inclusions of dendritic titanomagnetites were self-assembled and formed from exsolution within host silicates^53^. Nano-sized titanomagnetite inclusions can occur as randomly oriented particles within silicates whereas the sediment mixture also contained irregularly shaped, micron to submicron-sized titanomagnetites particles that likely resulted from the erosion of igneous rocks^53^. Prior work also confirmed the presence of self-assembled magnetic nanoparticles as titanomagnetites with variable Ti contents using HRTEM, SAED, and TEM-EDXS analyses^53^. Nanosized magnetite inclusions have been described in clinopyroxene and plagioclase^58^, with micro-inclusion habits of needles or elongated plates^59^, and as magnetite-orthopyroxene intergrowths associated with olivine in a Martian meteorite^60^, to name a few. In agreement with recent work, we also observed multiple forms of titanomagnetite in the grain environment that appear to be inclusions within the glass matrix rather than a product of microbial activity (Figs. 3, 4, 5, 6).

APT has been increasingly applied in the Earth sciences to address how rocks and minerals transform, evolve, or decompose over time by offering near atom-scale resolution of the elements comprising geological materials^37^. Additionally, prior work employed APT to investigate the atomic-scale mechanisms that control the alteration of glass over multiyear to decadal timescales (e.g., 2 to 25 years) in controlled laboratory experiments^10,61,62^. Magnetite particles have been extensively studied with APT including work on fabricated magnetite^63^, organic–inorganic interfaces in chiton teeth^64^, or magnetite from volcanic ash deposits^65^. The APT data in our study suggested a subtle enrichment of Ca at the glass-titanomagnetite contacts in both grains as evidenced by the 3D elemental distribution data (Figs. 8, S10), which revealed abrupt transitions among the mineral phases for Fe, Mn, and Ti versus less abrupt boundaries for Ca and P (Fig. 8). This is the first effort to use APT to provide a three-dimensional representation of the elements distributed across a basaltic glass-titanomagnetite interface following exposure to soil field conditions. Laboratory experiments revealed that Ca- and P-containing whitlockite, a mineral similar in composition to apatite, accumulated as small grains in compositional boundary layers at the margins of magnetite and pyroxene in ferrobasalts^66^. APT was also combined with SEM and electron microprobe analysis to identify Fe-rich compositional boundary layers at melt-crystal interfaces in basaltic glass from Hawaii, the Snake River Plain, and Iceland^67^.

### Enrichment of Ca at the glass-titanomagnetite interface

The nanoscale enrichment of Ca along the basaltic glass-titanomagnetite contact was likely preserved in the pristine glass during the formation of the basalt (Fig. 8). To our knowledge, this is the first time a Ca enrichment layer has been visualized along basaltic glass-titanomagnetite interfaces at the atomic scale. We attribute the Ca layer to ion diffusive (i.e., Leisegang-type) processes that occurred during the formation of the titanomagnetite inclusions in the basaltic glass given the small, subtle nature of the enrichment and that the enrichment occurred over a short distance on the order of < 5 nm in both instances (Fig. 8). Constraining the mechanisms of formation (i.e., supersaturation, nucleation, and growth) for the Ca enrichment are outside the scope of this study; however, we predict that the enrichment process occurred during the growth of the magnetite crystals in a cooling silicate melt, and that the enrichment has since been preserved in the basaltic glass. We note that the TEM analyses did not reveal evidence for the precipitation of Ca secondary minerals or more prevalent Ca enrichment occurring along the grain surface or the interfacial boundary in either grain (Figs. 8, S4). The Ca enrichment was likely too low in concentration to detect using TEM analyses. Additional interfaces need to be analyzed, but we hypothesize that the concentration of Ca in the thin enrichment layers in combination with the enhanced alteration of basaltic glass in the presence of magnetite creates a more advantageous environment for fungal foraging and mining activities compared to pristine, unaltered glass that has not been weakened by magnetite inclusions (Figs. 8, S10–S12).

We compared our interpretations of Ca enrichment in the present work with data from nuclear glass alteration and radioactive waste simulation experiments where APT has been employed extensively^9,10,68,69^. However, the design of the nuclear glass alteration experiments created intensive weathering regimes in controlled settings compared to the field conditions in our study, specifically with respect to time, temperature, degree of silica saturation, among other factors. For example, one set of glass dissolution experiments documented the continued presence of mobile elements (i.e., Ca, Na, B) in a thin, passivating alteration layer on glass coupons reacted under initially Si-saturated conditions for 875 days at 90 °C and pH 9^10^. The presence of magnetite, an Fe-corrosion product pertinent to the storage of nuclear waste, enhanced the dissolution of nuclear glass as documented in work that mixed nuclear glass and magnetite powders and subsequently described the formation of Fe-enriched gel alteration layers that thickened with time^68^. Glass-iron-clay laboratory experiments designed to simulate the geological disposal of radioactive waste also presented glass-magnetite/siderite/Fe-rich phyllosilicate interfaces that documented the formation of a protective gel layer and a secondary precipitates layer that concentrated Ca, P, lanthanides, and Mo^9,69^.

### New perspectives on incipient weathering

This research provides new nano- to microscale perspectives on the initial stages of alteration of basaltic grains exposed to humid, wet conditions in a natural soil system. We observed the growth of fungal hyphae across grains and assessed the resulting interfaces to advance understanding of microbe-mineral interactions over a three-year time scale. Our work emphasizes the complexity associated with differentiating biotic from abiotic processes in field environments even with the use of nylon mesh bags to deploy unreacted granulated rock substrates. We observed fungi inhabiting grain surfaces and edges including a fungal hypha embedded in an interwoven mat or sticky biofilm coating. We refuted an initial hypothesis that the magnetite detected on a basaltic grain surface formed from biomineralization given its proximity to a fungal hypha and the absence of magnetite observed on other grain surfaces with no fungal growth (Figs. 2c, 7). Instead, we directly observed that the titanomagnetite crystals are inclusions within the basaltic glass matrix, and we also identified evidence for the incipient stages of glass alteration near the grain surface (Fig. 5). Our work highlights the need to examine grains with a multi-faceted approach given the importance of vertical cross-sections for showing potential dissolution fronts, alteration features, and enrichment layers across interfaces (e.g., fungal-grain, glass-titanomagnetite). We conclude that the changes to the grain surface at the fungal contact are likely biotic in nature given that the concave shape occurred immediately below the grain contact with the fungal hypha (Fig. 5).

Fungi are known foragers that selectively mine nutrients from minerals and do so by expending as little energy as possible in the process^21,46^. Basaltic glass represents an energetically favorable source of growth-supporting nutrients (i.e., Ca, P, Fe) compared to crystalline minerals that are less susceptible to chemical weathering (Table S1). We predict that the fungi in the present study were attracted to the basaltic glass grains with titanomagnetite inclusions given that magnetite enhances glass alteration^68^ and serves as a source of iron, an essential micronutrient, to microbes^70^. Therefore, the presence of titanomagnetite on the surface or near-surface of basaltic grains may make the glass-titanomagnetite interface more accessible for fungal nutrient uptake versus areas of the basaltic glass containing no titanomagnetite inclusions exposed on or near its surface. We acknowledge that our interpretations are based on two fungal-grain contacts and that more assessments are required to understand fungal foraging activity across basaltic grains, and whether there is a preference for glass-titanomagnetite interfaces. However, our findings suggest that the fungi may have exhibited opportunistic growth and interactions with regions of the basaltic glass matrix containing Fe-bearing inclusions which are exposed on or very near basalt grain surfaces.

Prior work demonstrated that fungi can act as biosensors by allocating energy to grow proximal to P-containing apatite over other mineral types^21^ and lead to the amplified release of P and Ca from basaltic materials in laboratory experiments^71^. Soil Fe from Fe-bearing minerals can be used in electron transfer processes either as an energy source by chemolithotropic microbes (Fe^2+^ → Fe^+3^ + *e*^*−*^) or as terminal electron acceptors by heterotrophic microbes (Fe^+3^ + *e*^*−*^ → Fe^2+^) under dysoxic, or low oxygen, conditions that are known to occur in the upland Ultisols of the Calhoun field site^72^. Siderophores, or “organic compounds that bind (chelate) and transport Fe to microorganisms”^23^, are also produced by microbes (i.e., fungi, bacteria, lichen) and have been associated with enhancing molecular-scale interactions between surfaces of Fe-rich minerals (i.e., goethite, lepidocrocite) and the siderophore azotobactin^73^. Evidence for biogenic etching and the bioalteration of basaltic glasses through the siderophore-mediated release of Fe has also been observed for multiple strains of bacteria and fungi in laboratory studies^26,74–77^.

### Heterogeneity of processes at the microscale

**Figure 9:**
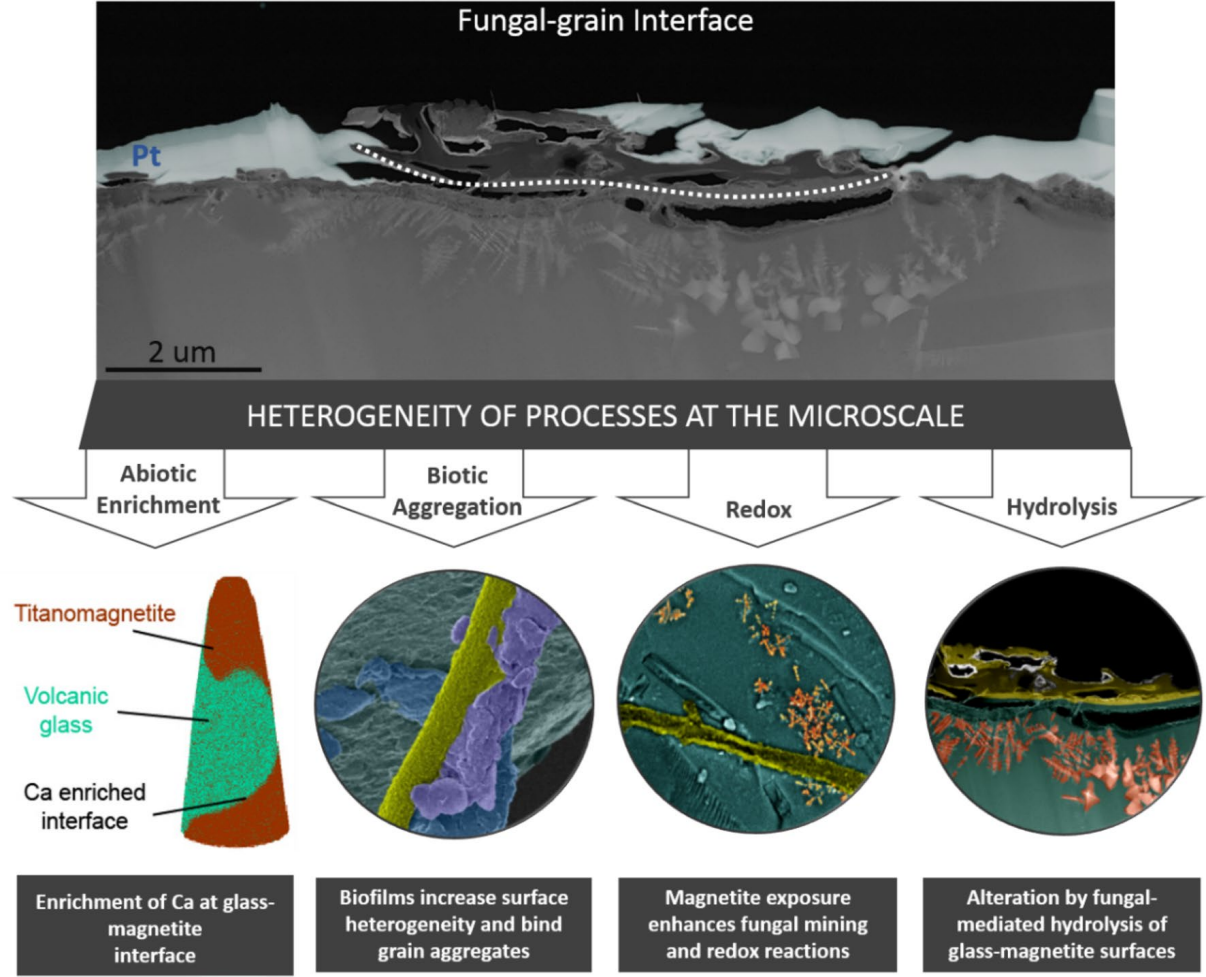
A summary schematic to conceptualize the heterogeneity of abiotic and biotic processes at the microscale that were identified as direct or indirect observations in our study.

We present a conceptual model to demonstrate the heterogeneous nature of We present a conceptual model to demonstrate the heterogeneous nature of fungal-grain interactions at the sub-micro scale and the importance of employing a multifaceted approach when examining microbe-grain interfaces (Fig. 9). By direct or indirect observations, our study acknowledges these potential processes and reactions as demonstrative examples:

i. *Abiotic Enrichment.* The enrichment of Ca at the glass-titanomagnetite interface represents an abiotic process likely resulting from the Liesegang phenomenon or pattern formation defined as “the appearance of the nonhomogeneous spatial distribution of the concentration of one or more chemical species”^78^. The abiotic enrichment of nutrients has important implications for assessing microscale controls on elemental cycling and mineral transformation processes.
ii. *Biotic Aggregation.* The 3-D arrangements and interactions of soil mineral particles and organic matter compounds in soil aggregates exert significant control on organic matter mineralization, the persistence of soil carbon, and elemental cycling in soils^79–82^. Filamentous fungi promote the formation of aggregates in soil by entangling soil particles in hyphal networks or secreting exudates that cement or seal the surfaces of aggregates^83–85^. The fungi inhabiting grain surfaces in the present study contained fungal hyphae embedded in an interwoven mat, sticky coating, or biofilm that appeared to be contributing to initial stages of particle aggregation (e.g., Fig. 1d).
iii. *Hydrolysis.* There is visual evidence for fungal-driven dissolution of the basaltic glass given the 1-µm deep concave shape identified directly at the fungal-grain contact that is not visible elsewhere along vertical cross sections that are unoccupied by fungi (Figs. 5, 7, S9). We suggest that the incipient weathering observed in our study may result from biosensing, nutrient mining, and acquisition activities by fungi as shown in a mesocosm study that displayed evidence for resource foraging by mycorrhizal fungi (i.e., 21). Biological weathering features were also identified in the form of channels or trenches on chlorite, biotite, and hornblende during laboratory studies focused on fungal-mineral interactions in microcosm experiments^86,87^.
iv. *Redox Processes.* We hypothesize that the fungi contributed, at least in part, to the glass dissolution mechanisms that led to the broader exposure of titanomagnetite crystals on the basaltic grain surface. Volcanic glass is more energetically favorable to weathering compared to exposed mineral phases, such as magnetite or feldspars that are more resistant to weathering (Fig. 2^88–90^). The exposure of magnetite on grain surfaces has important environmental implications given that magnetite represents a redox-sensitive, Class III mineral with a tendency towards high structural breakdown and molecular growth^91^. Interactions between adsorbed organic matter and magnetite are expected to modify the surfaces of both the organic matter and mineral phases^91^ and demonstrates the need to better understand possible redox processes between magnetite and sensitive organic phases in natural field settings.

### Implications

Ongoing microscale investigations of fungal-grain interfaces and mineral weathering processes are required to advance the fundamental understanding of our natural and built environments. Basalt weathering releases rock-bound nutrients to ecosystems, consumes atmospheric carbon dioxide that becomes subsequently stored in reservoirs of carbonate rock over geologic time, and remains an important area of research required to better understand how microbes inhabited basalt to establish life on early Earth and possibly on other planetary bodies. Importantly, a wide breadth of societal challenges align with unanswered questions pertaining to basalt and glass weathering including how biota may influence or transform rock and mineral materials. For example, how does (a)biotic mineral weathering drive nutrient cycling, the persistence of soil carbon, and soil formation in terrestrial environments on Earth and beyond? What mechanisms will securely preserve spent nuclear fuel to ensure the safe, geological storage of radioactive waste? How can we best preserve and conserve glass artifacts, such as medieval stained glass, or other cultural heritage materials? These questions reiterate the pressing need to understand how weathering processes in the field differ from simulations or controlled settings in the laboratory given the complex, intricate nature of field environments.

## Methods

### Field setting and experimental design

Our study assessed fungal-grain interactions in basaltic material from a larger incipient weathering project that deployed granulated rock substrates (i.e., basalt, granite, quartz sand) along a regional climate gradient for 1 to 3 years^41^. The unreacted basalt substrate was originally collected from the Merriam crater (Flagstaff, AZ) and underwent extensive processing, cleaning, and characterization prior to use in laboratory and field weathering experiments^50^. The granulated substrates (250–53 µm) were heat sealed into nylon mesh bags, autoclaved, and buried in the upper 10 cm of mineral soil horizons. The basaltic grains analyzed herein were deployed in a fine nylon mesh bag (0.5 µm mesh size) and retrieved following three years of burial in a mixed hardwood forest site, with a mean annual precipitation of 127 cm year^−1^ and a mean annual temperature of 16 °C (United States Forest Service Calhoun Experimental Forest, South Carolina). The mixed hardwood-pine forest soils formed on granitic gneiss (biotite-quartz-feldspar mineralogy) from the Whitmore Complex where quartz and amphibolitic dikes and lenses were also described^92,93^. Hardwood tree species in the field area range from Shortleaf Pine (*Pinus echinata*), N. Red Oak (*Quercus rubra*), and Sweetgum (*Liquidambar styraciflua).*

We selected basaltic rock substrate for our work given that basalt supplies fine-grained mineral inclusions and elemental nutrients in a basaltic glass matrix that is more susceptible to weathering compared to granite^13,27^. The basalt substrate contains nutrients within the basaltic glass matrix (i.e., Ca, Mg, K, Fe, P; see Table S1), pyroxene (i.e., Ca, Mg, Fe), feldspar (Ca), and olivine (i.e., Mg, Fe), among others^50^. The basaltic glass is a dominant solid phase comprising 40% ± 2.5% of the basaltic materials, with inclusions of plagioclase feldspar (34% ± 2.0%), the pyroxene augite (11% ± 1.1%), quartz (0.4% ± 0.2%), the Mg-rich olivine forsterite (14% ± 3.7%), and magnetite (0.5% ± 0.1%) as determined by X-Ray diffraction (Table S2). Electron microprobe elemental analyses of the basalt substrate conducted in a previous study suggested the presence of feldspar, augite, forsterite, and minor amounts of chromite and apatite embedded in the basaltic glass matrix^50^. Prior work also identified the presence of micron-sized titanomagnetite inclusions in the basaltic glass (~ 3 µm^50^).

### X-ray diffraction (XRD)

XRD analysis was used to characterize the unreacted basalt using a Philips X'Pert MPD system with a vertical Bragg–Brentano goniometer on randomly oriented powder mounts, with an internal standard (Al_2_O_3_) for phase quantification. The X-ray source was a long-fine-focus ceramic X-ray tube with a Cu anode operated at 45 kV and 40 mA. Data were collected using a variable divergence slit between 5 and 100° 2θ with a scan step of 0.04° at a rate of 0.6° 2θ per minute. Quantitative compositions were determined by whole-pattern Rietveld fitting^94^ of the XRD patterns using TOPAS v4.2 (Bruker AXS). Allowing for minimization of structural factors including lattice parameters, intensity, coherent scattering domain size, and in some cases, preferred orientation bias, final model results were achieved with typical weighted profile residual (Rwp) ranging from 10.8 to 13.1%. The mineral fractions were scaled to the internal standard and the difference between the sum of minerals and 100% was ascribed to amorphous material.

### Focus ion beam/scanning electron microscopy (FIB/SEM) and transmission electron microscopy (TEM)

We identified microbe-grain interfaces on grains subsampled from the basalt substrate after three years of exposure to biotic inputs in the same field area studied previously^41^. Here, we reference the fungal-grain interfaces in association with “basaltic grains” based on the understanding that the grains encompass a basaltic glass matrix and fine crystalline inclusions. Our investigations focused on two basaltic grains, referenced throughout as Grain 1 and Grain 2, that were selected to compare vertical cross sections of two fungal-grain interfaces along the surface (e.g., Grain 1) and edge (e.g., Grain 2) of samples with a basaltic glass matrix. Grain 1 was approximately 150 µm in length and contained inclusions visible on the grain surface adjacent to the fungal contact (Figs. 2, 3) whereas Grain 2 was approximately 100 µm in length and showed no evidence for the exposure of inclusions on its surface (Fig. 2).

General SEM surveys of Grain 1, Grain 2, and three additional basaltic grains referenced as Grains A, B, and C were performed to identify microscale weathering features and microbe-grain interactions, to seek evidence for biotic inputs to the mesh bag samples, and to select fungal-grain interfaces for more intensive microscopy investigations (Figs. 1, S1). We selected Grains 1 and 2 for the intensive FIB/SEM/TEM investigations to contrast the nature of fungal-grain contact and crystalline minerals on the grain surface (i.e., Grain 1) versus Grain 2 where no surficial minerals or inclusions were visible adjacent to the fungi. The scope of our study only included analyses of fungal-grain interfaces on Grains 1 and 2 due to the complexity and expense associated with the sample preparation and analytical techniques.

The FIB/SEM and energy dispersive X-ray spectroscopy (EDX) analysis was performed using a FEI Helios NanoLab 600i field emission electron microscope. SEM analyses were performed after initially coating the samples with ~ 10–15 nm of carbon using a thermal deposition C-coater to minimize imaging artifacts due to charging. High resolution secondary electron images were collected at an accelerating voltage of 3 kV and beam current of 0.086 to 0.17 nA using immersion mode and through-the-lens (TLD) detector. SEM images were collected at a working distance of 3.5–4 mm. The EDX analyses were conducted using 10–20 kV voltage and 1.4 nA current using a solid state detector (SSD) with 80 mm window; this configuration improves the precision of EDX analysis for samples with rough morphology. We employed SEM/TEM and the respective EDX analyses to assess organic and inorganic materials within the samples by examining morphological features and performing elemental analyses. The EDX analytical technique enabled us to distinguish carbon-rich, organic-based materials (i.e., micron-sized fungal structures and other micron-sized soil microbes) from inorganic mineral particles containing Si, Al, Fe, Ti, or other elements. We used morphological assessments at the nanoscale when the analytical capabilities were unable to distinguish the elemental distribution, i.e., in nanosized particles that were adhered to fungal or other microbial structures.

Three cross sections of fungal-grain interfaces were exposed on Grain 1 and Grain 2 using a FIB Ga liquid metal ion source milling and lift-out technique in order to prepare thin lamellas for TEM analysis. A limited number of two TEM sections were studied due to the difficulty and cost of preparing appropriate, wide-area, and properly thinned areas with the latest FIB technology. Prior to ion milling, the areas of interest were protected by the deposition of a 1–2 µm Pt layer using Helios GIS (gas injection system). The specimens were thinned to 80–100 nm by using lower beam currents to below 100 pA. The vertical cross sections enabled us to assess the morphological properties of the basaltic glass matrix, the mineral inclusions, and the fungal-grain contacts. TEM analyses were then performed to identify the composition and elemental abundance of the basaltic glass matrix and mineral inclusions. TEM imaging and analysis were carried out with a FEI Titan 80–300 microscope operating at 300 kV. Imaging was performed using a high-angle annular dark field (HAADF) detector in the STEM mode. We also used conventional broad beam imaging and selected area electron diffraction (SAED). Analysis of diffraction patterns was performed using Gatan Digital Micrograph. The images and diffraction patterns were recorded using Gatan’s UltraScan1000 2 k × 2 k charge-coupled device (CCD) camera. Compositional analysis was performed with EDX, using an Oxford X-MaxN100TLE solid drift detector SDD (100 mm^2^). The EDX data collection and processing were achieved with Oxford’s Aztec software package.

### Atomic probe tomography (APT)

APT specimens were extracted approximately 15 to 20 µm from the fungal contact in Grain 1 including one tip from the glass-titanomagnetite boundary (e.g., Fig. S13) and a second from the basaltic glass matrix for comparison. We also extracted 3 tips immediately beneath the fungal-grain contact in Grain 2 (i.e., Fig. 5a) with two tips encompassing the basaltic glass-titanomagnetite boundary and the third entirely from the basaltic glass matrix. The specimens were sharpened into needle shapes with tip diameters < 100 nm through FIB established lift-out techniques^49^ using dual beam FIB/SEM. Specimens were extracted from within ∼1 µm of the area of interest. APT analysis was conducted using a Cameca LEAP 4000X-HR atom probe instrument, with the specimen temperature at 44 K. Thermally-assisted field evaporation resulting in a detection rate of 1000 ions s^−1^ was achieved with a 355 nm UV laser set at 50 pJ energy focused on the specimen tip and pulsed at 200 Hz. Data sets were reconstructed using Cameca’s Integrated Visualization and Analysis Software IVAS 3.8.4, using a tip profile approach guided by the SEM image of specimen tips. Mass spectral ranging was performed manually with mass-to-charge ratio ion assignments listed in Supplementary Table S3 and applied to the mass spectra for Grain 1 and Grain 2 shown in Supplementary Fig. S12.

## Supplementary Information

Supplementary Information.

Supplementary Video S1 - Phase 1.

Supplementary Video S2 - Phase 2.

Supplementary Video S3 - Phase 3.

Supplementary Video S4 - Phase 4.

Supplementary Video S5 - Phase 5.

Supplementary Video S6 - Phase 6.
