# Supplementary material for: Fungal hyphae develop where titanomagnetite inclusions reach the surface of basalt grains
Source: Sci Rep. 2022 Mar 1;12:3407. doi: 10.1038/s41598-021-04157-z (PMC8888555; doi:10.1038/s41598-021-04157-z)
Supplement: Supplementary file 1 — Supplementary Information. [file 41598_2021_4157_MOESM1_ESM.docx]

**Supplementary Information**


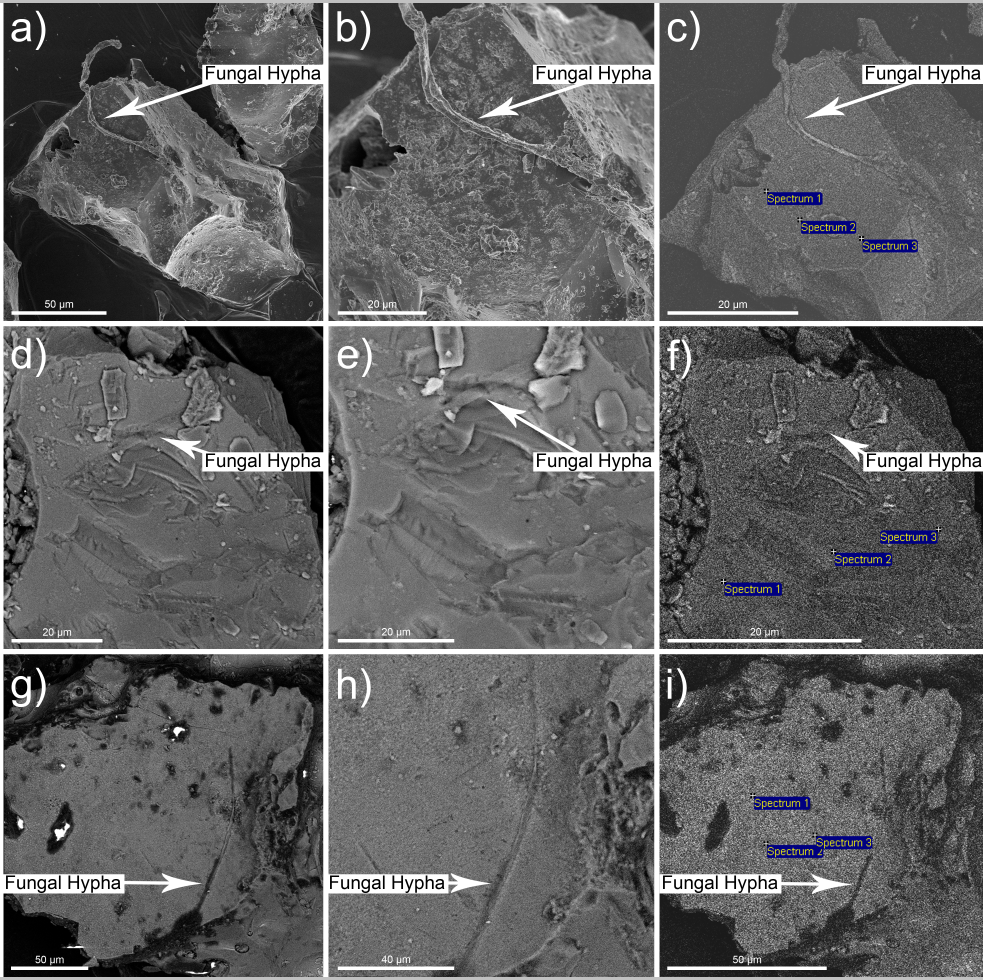


**Figure S1.** A survey of basaltic grains following three years of deployment at our field site (Calhoun Experimental Forest, SC) revealed fungal interactions with grain surfaces and edges as evidenced in electron images for a-c) Grain A, d-f) Grain B, and g-i) Grain C. The grains were comprised of a basaltic glass matrix that contains embedded crystalline minerals. The figure contains Secondary Electron (SE) images in a), b), d), e), g), and h) in addition to backscattered electron (BSE) images in c), f), and i).


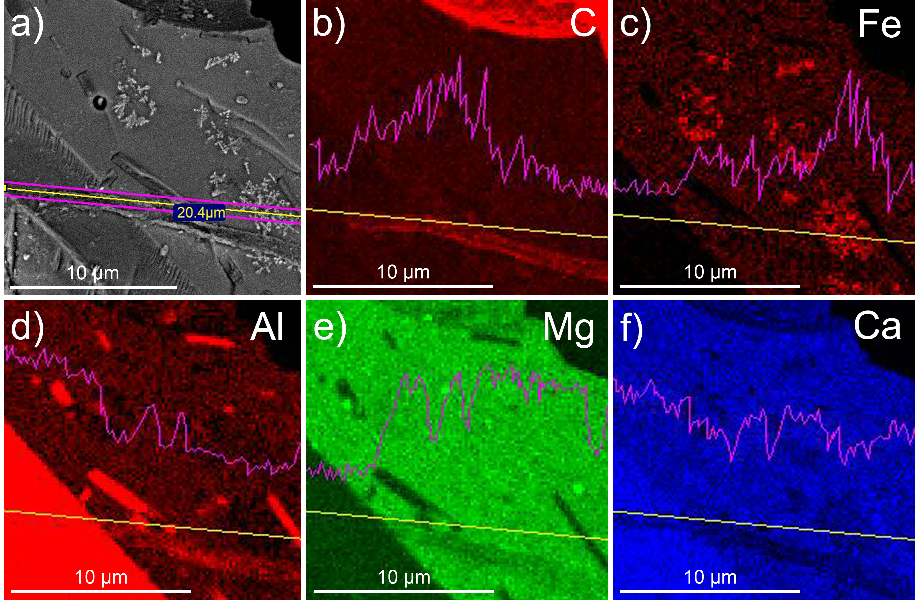


**Figure S2.** Elemental profile distribution on the surface of Grain 1. The data lines represent concentration profiles for each element of interest. The straight lines represent the line measurement analysis.


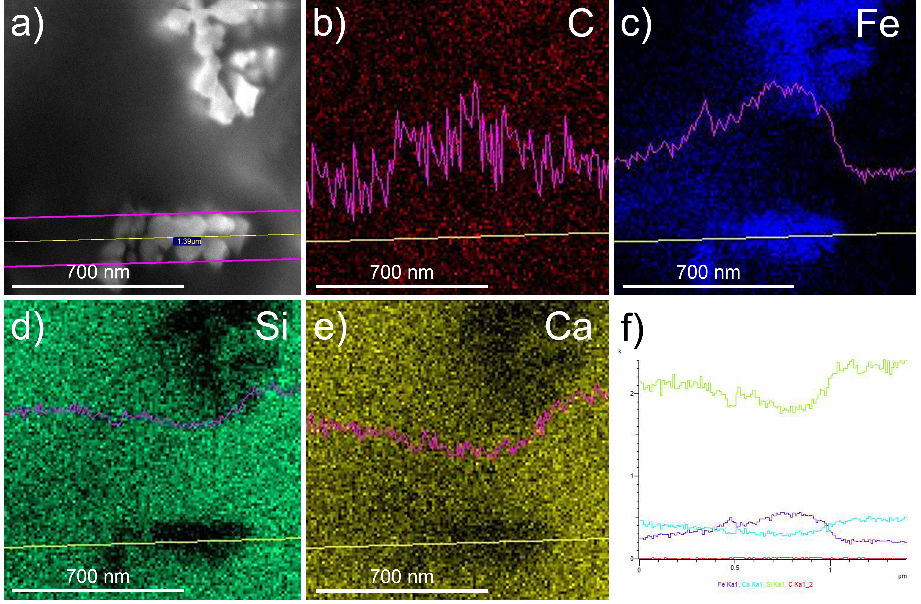


**Figure S3.** Elemental profile distribution below surface of basaltic glass matrix with embedded magnetite crystals in Grain 1. The data lines represent concentration profiles for each element of interest. The straight lines represent the line measurement analysis.


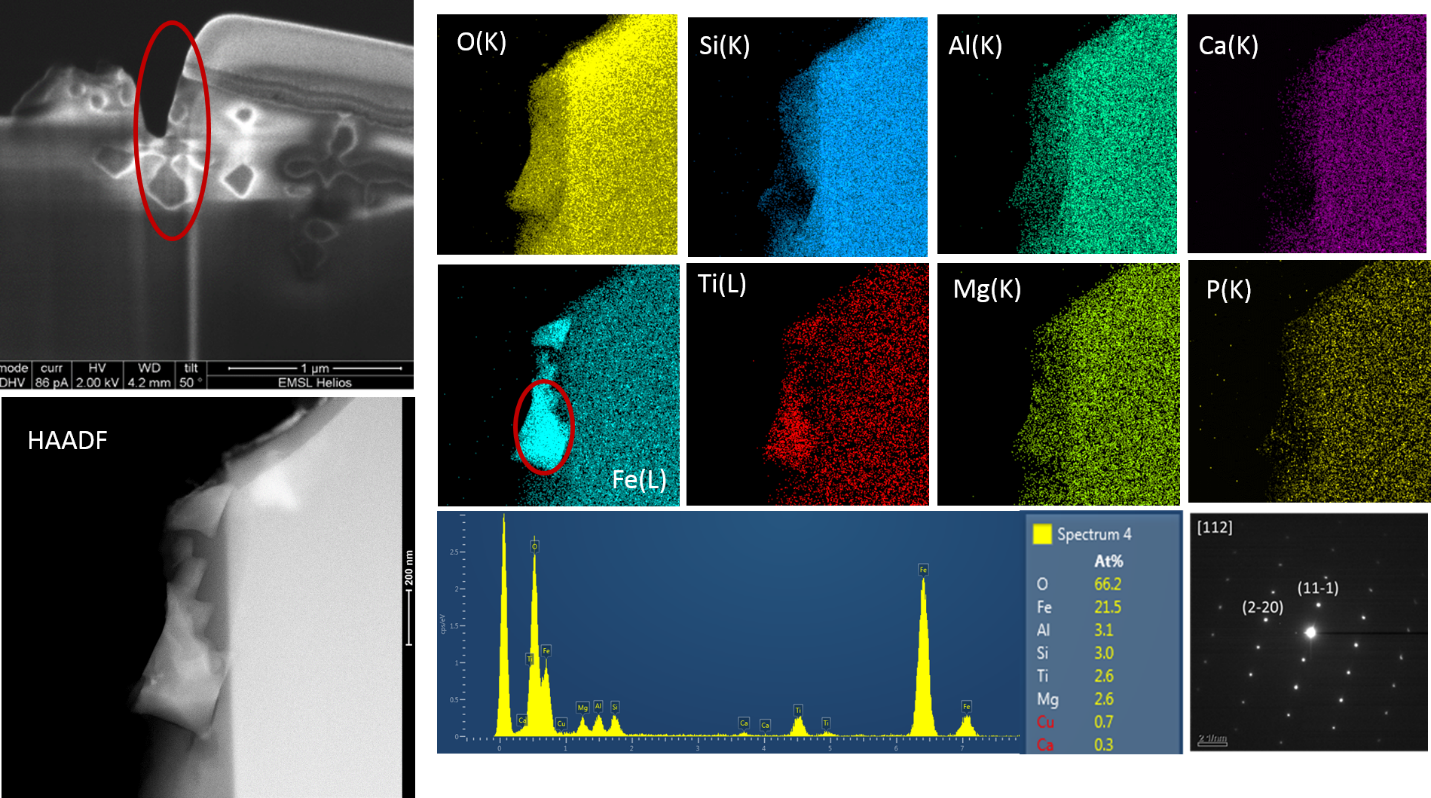


**Figure S4.** Identification of minerals adjacent to fungal hypha in Grain 1. Diffraction patterns are consistent with spinel structure of magnetite. EDX mapping and point analyses indicate a titanomagnetite phase.

*
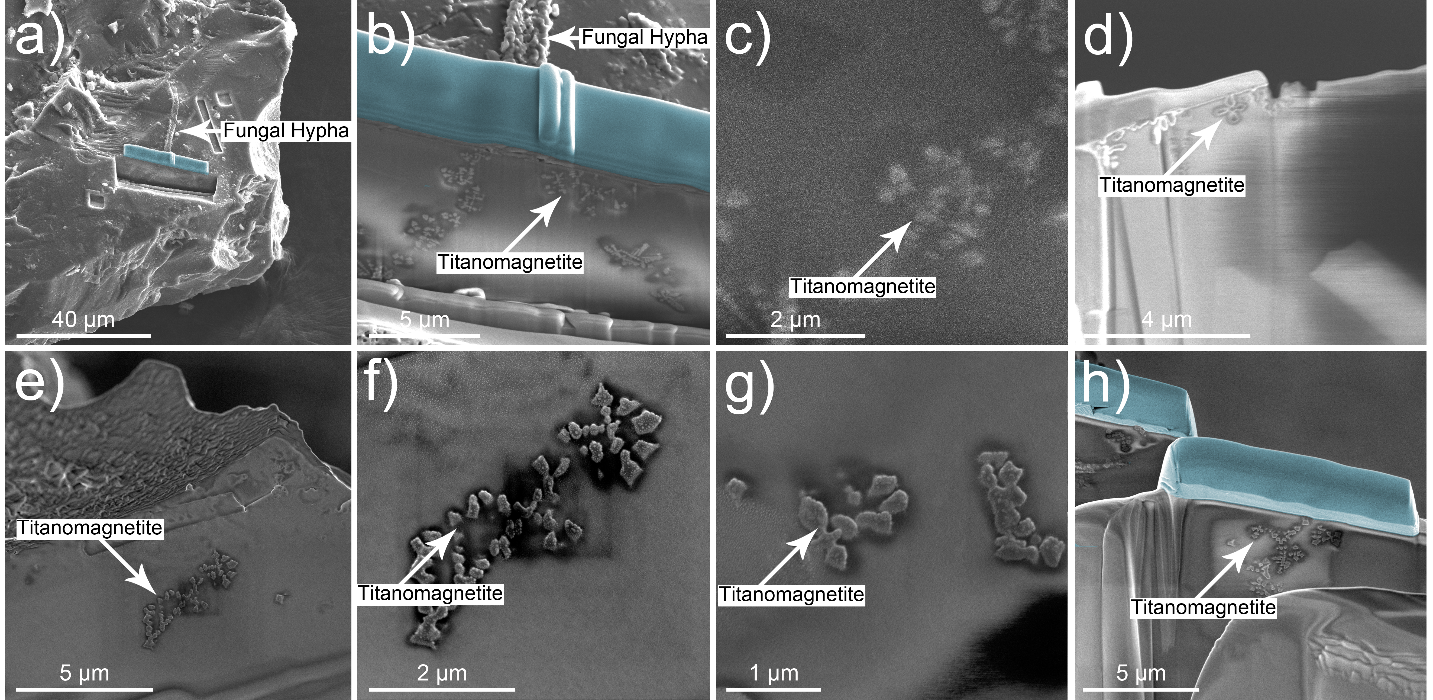
*

**Figure S5.** SEM micrographs at the surface and below Grain 1 surface. The Fe-rich particles occur on both sides of the fungal hypha, likely exposed due to dissolution processes.


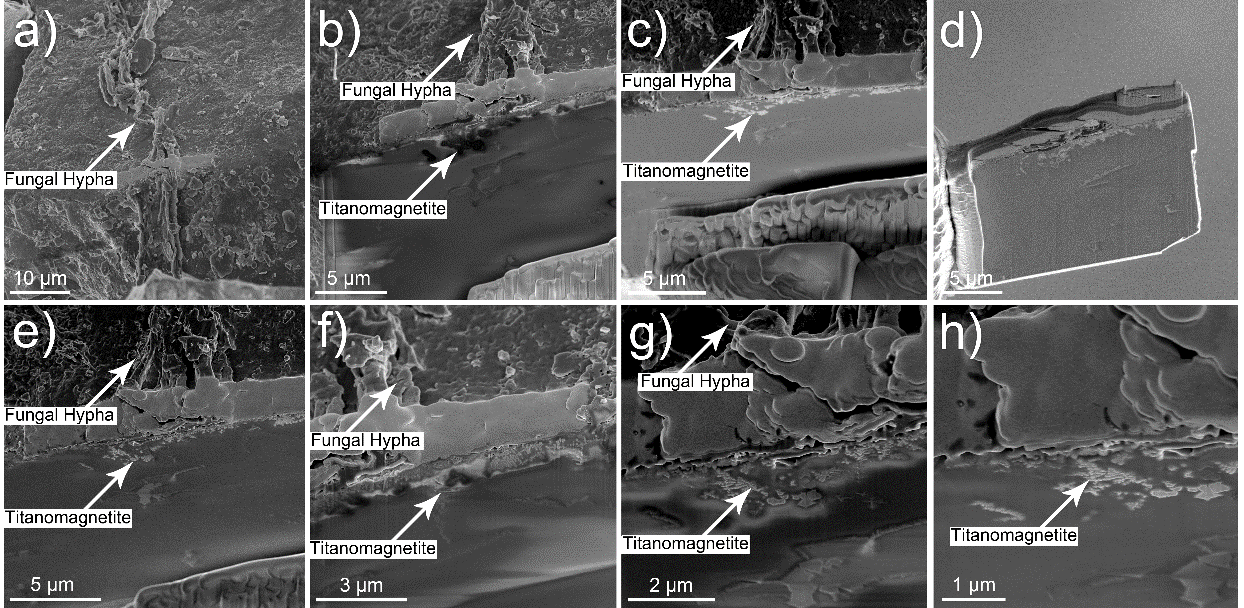


**Figure S6.** A series of SEM/FIB cross-sections for the front of Grain 2.


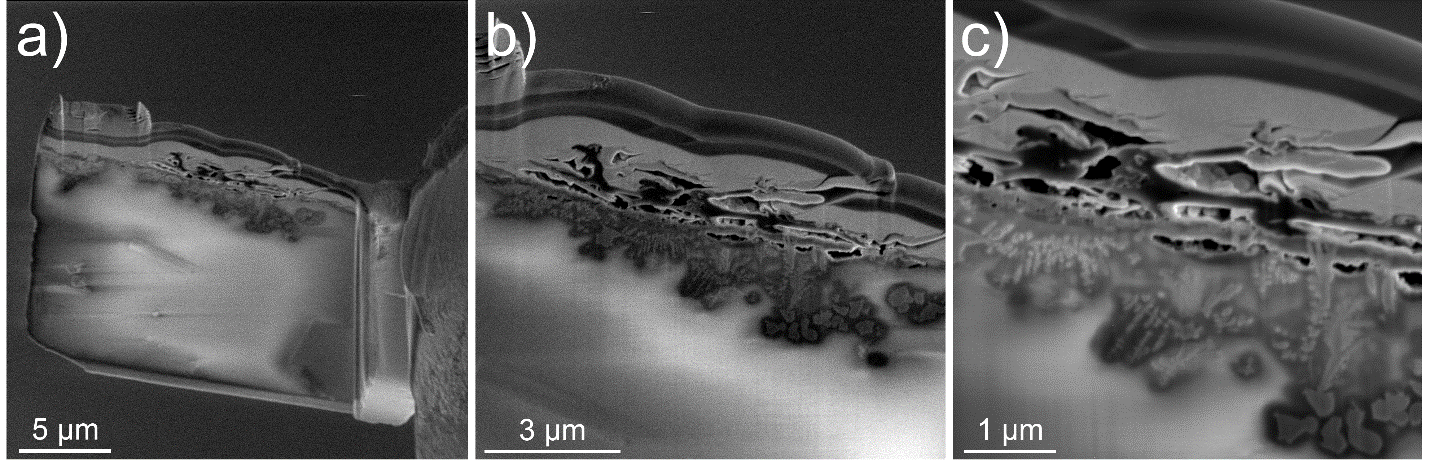


**Figure S7.** A series of SEM/FIB cross-sections for the back of Grain 2.


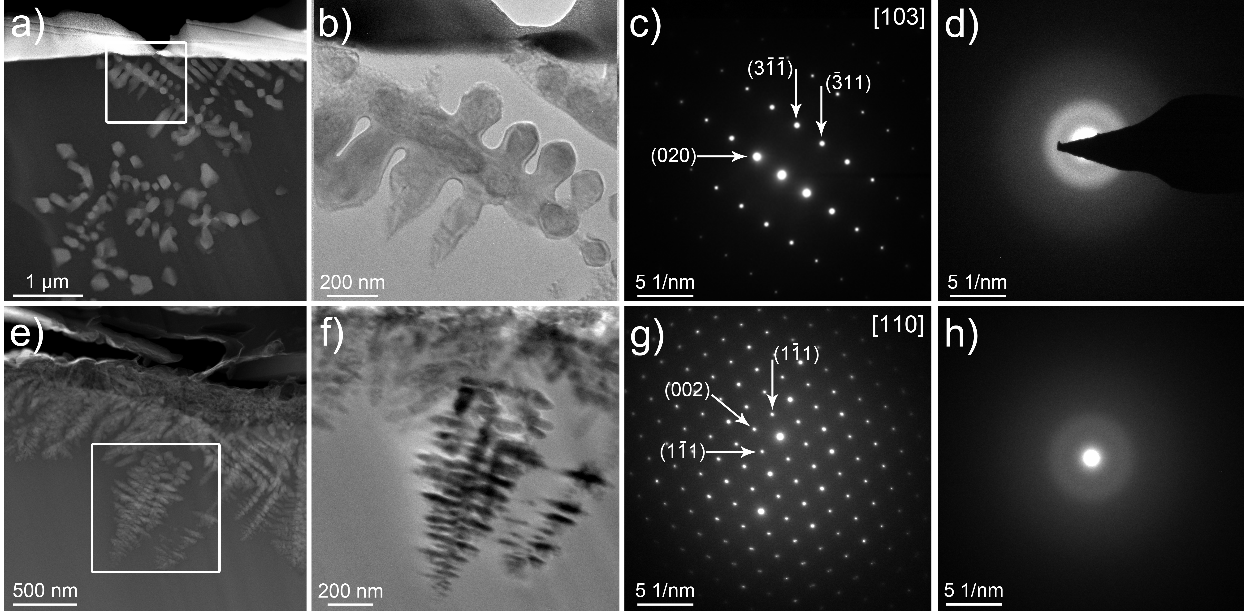


**Figure S8.** Diffractogram patterns indicating that host grains consisted of an amorphous basaltic glass matrix with embedded crystalline minerals. The diffractogram patterns were used to identify a-d) nanosize magnetite particles embedded in basaltic glass matrix from Grain 1 and e-h) dendritic titanomagnetite crystals along the fungal-glass contact in Grain 2.


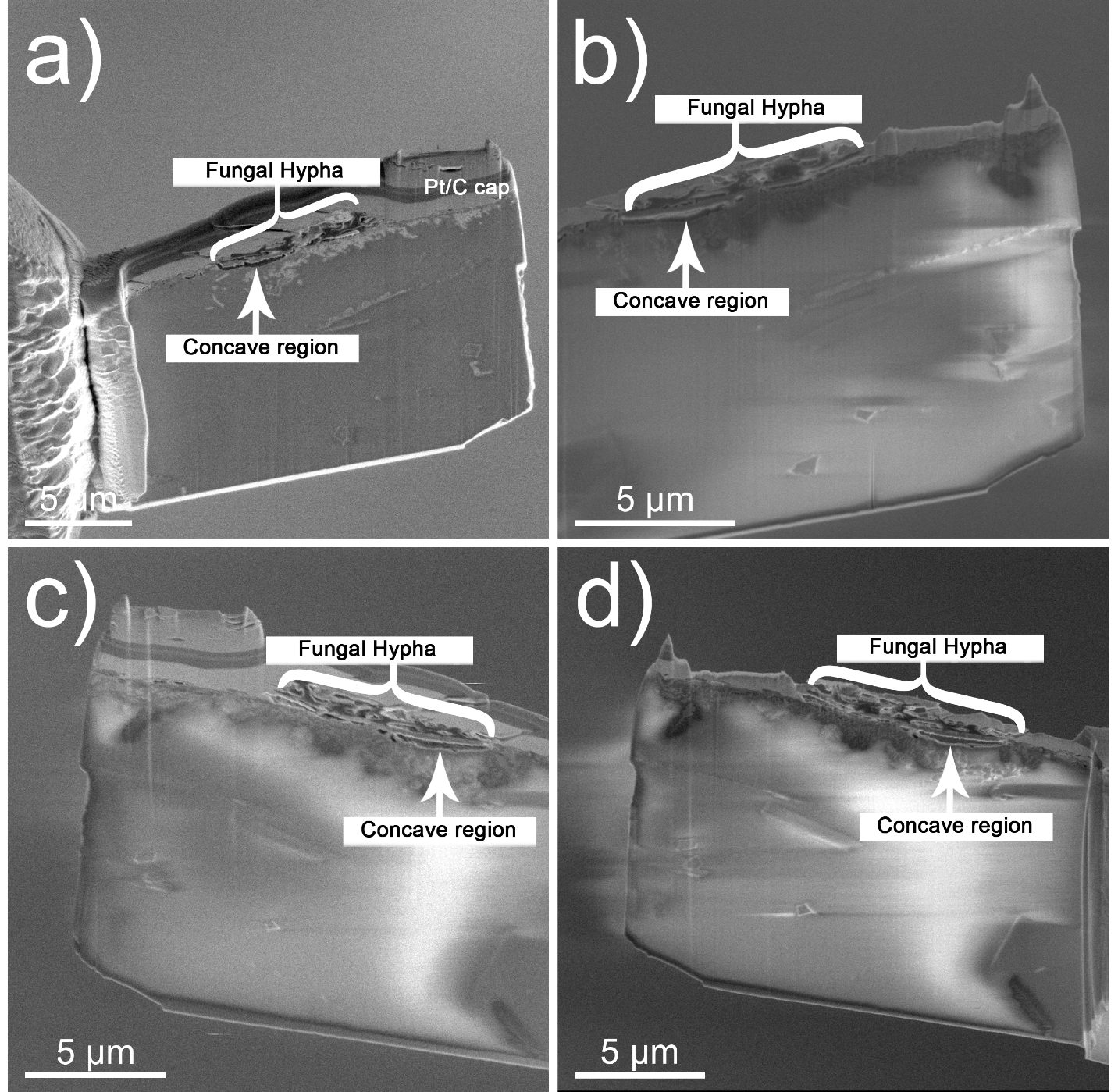


**Figure S9.** Sample preparation method for TEM analysis. The images show the a-b) front of the lamella including the concave feature immediately beneath the fungal-grain contact and the c-d) back of the lamella showing the preservation of the same features in the sample thinning and preparation process.


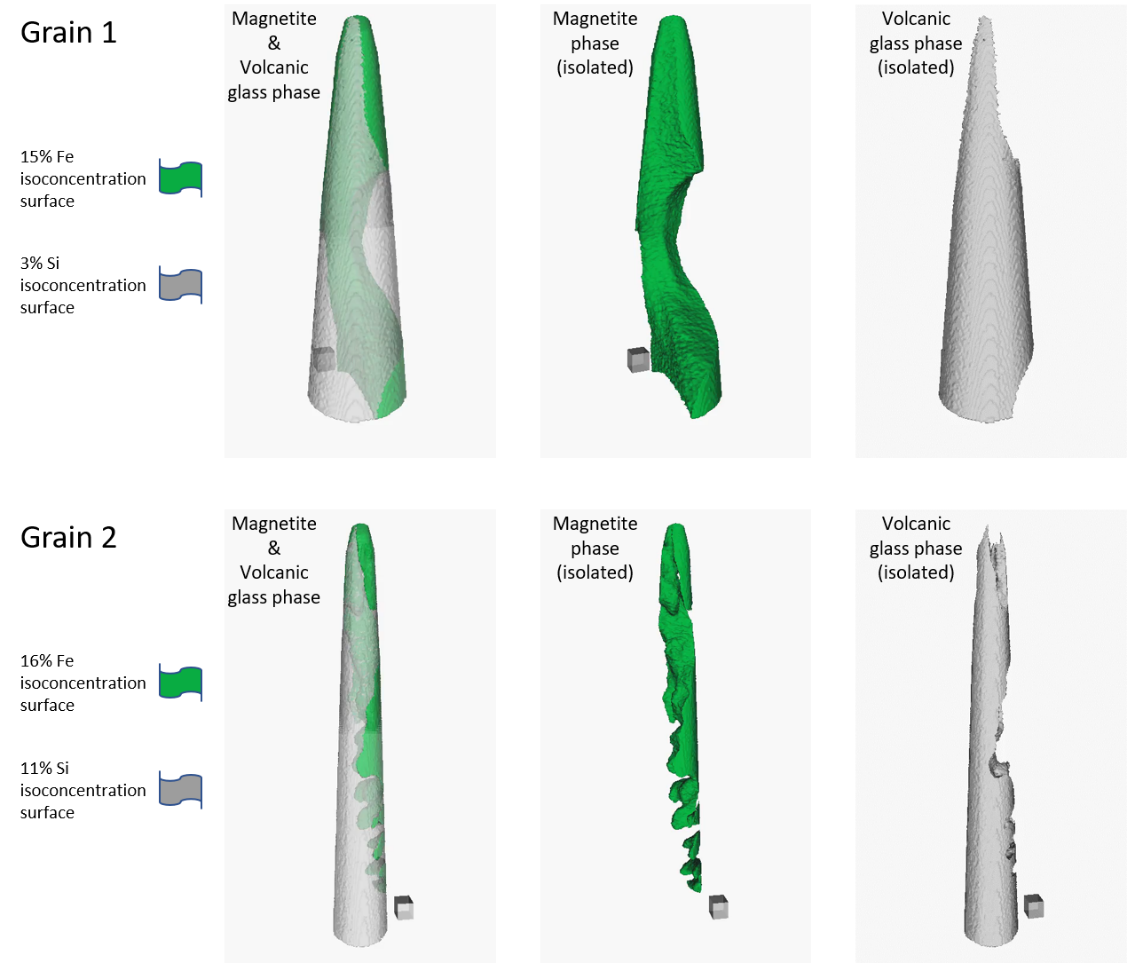


**Figure S10.** APT-based visualization of complex heterointerface morphology between basaltic glass matrix and titanomagnetite in Grain 1 and Grain 2. Supplemental animations are available for downloading and viewing each of the six phases shown here. Scale cubes are 20×20×20 nm^3^


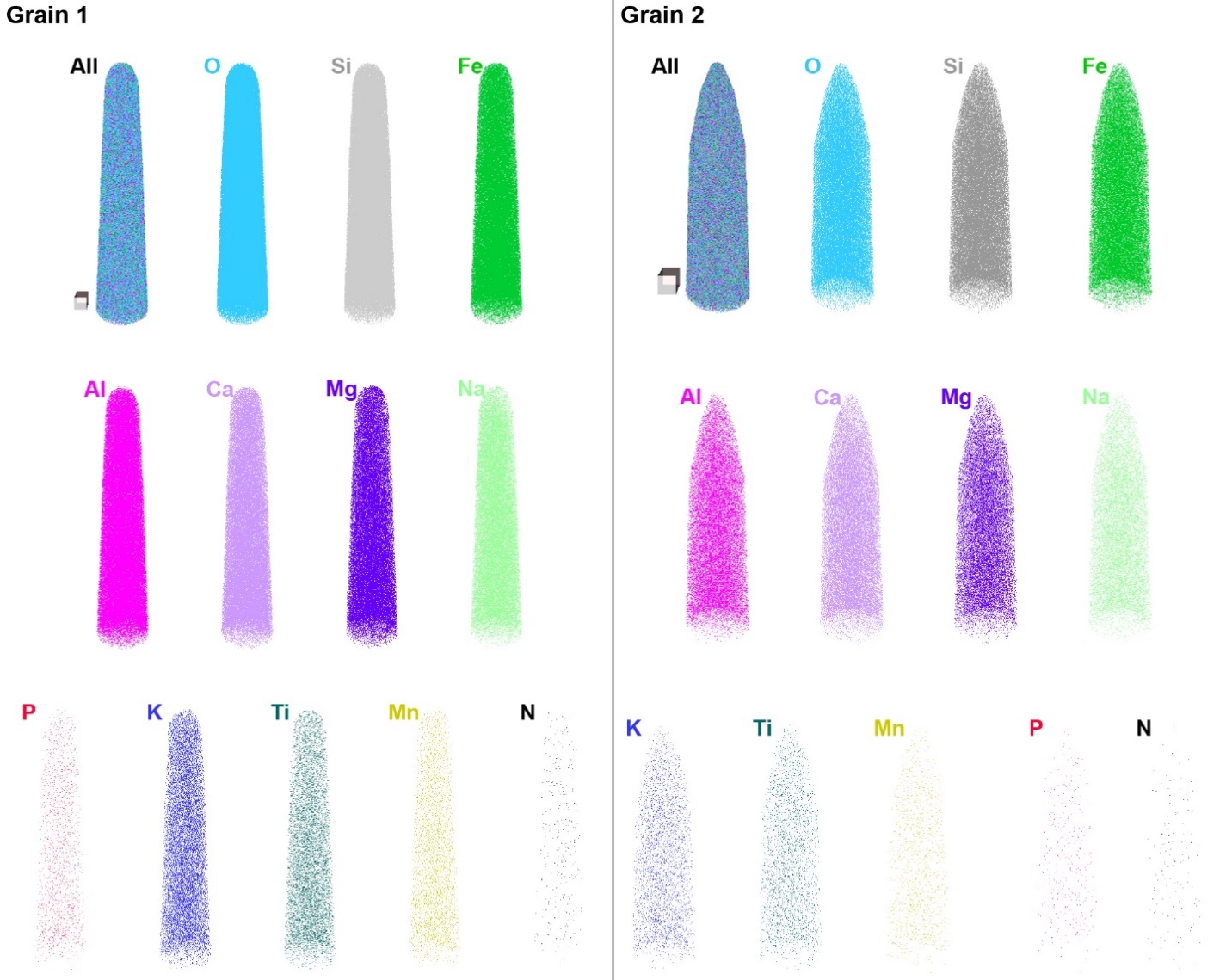


**Figure S11.** 3D elemental composition APT point cloud maps captured from within the basaltic glass phase of a) Grain 1 and b) Grain 2. Note that the analyzed volumes only include the basaltic glass phase (i.e., does not include bassaltic glass-titanomagnetite boundary). Scale cubes are 20×20×20 nm^3^.


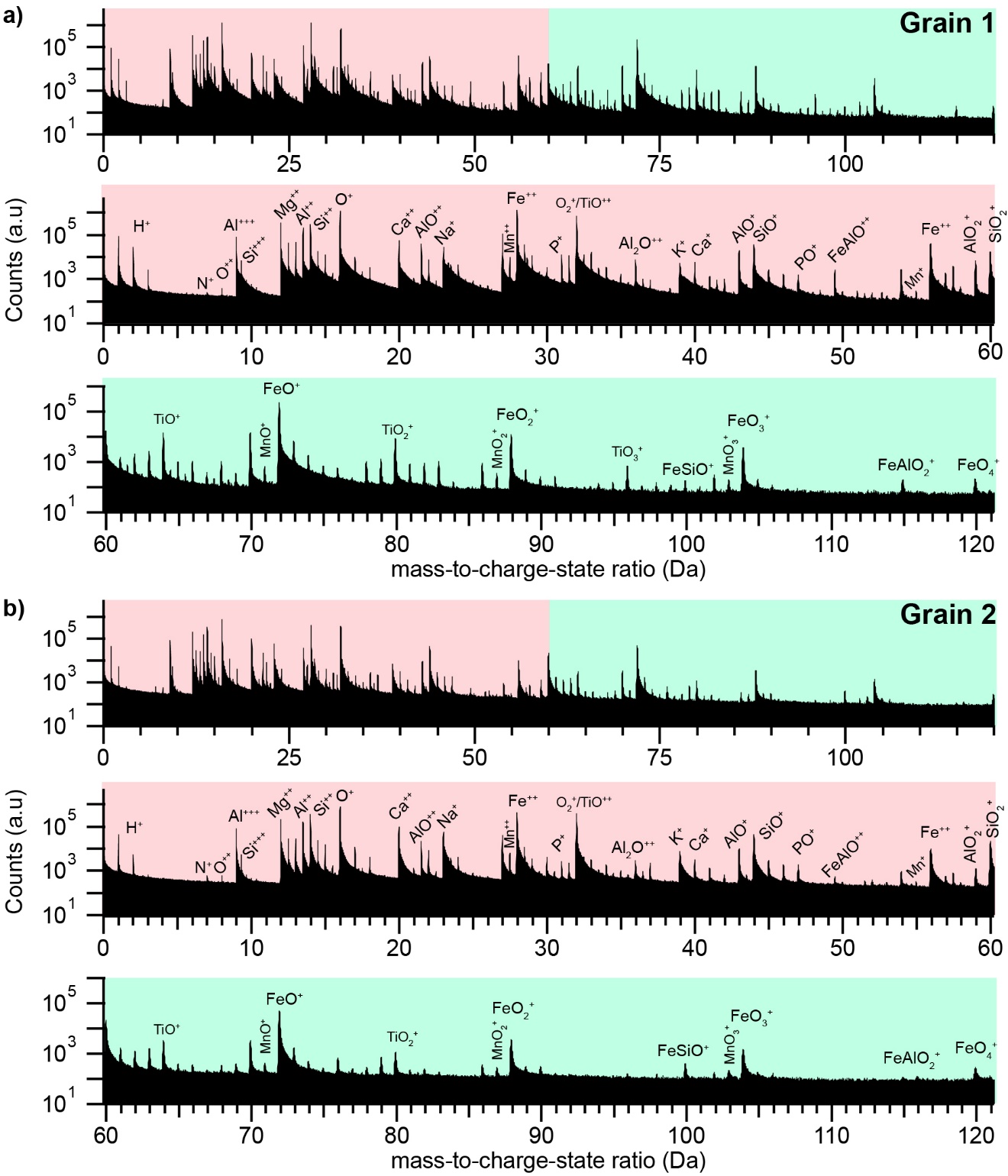


**Figure S12.** APT time-of-flight mass spectra of reconstructions shown in Figure 8 of the main text for a) Grain 1, and b) Grain 2. Magnified regions of selected mass ranges are color coded respectively. Mass ranging was performed manually within IVAS.


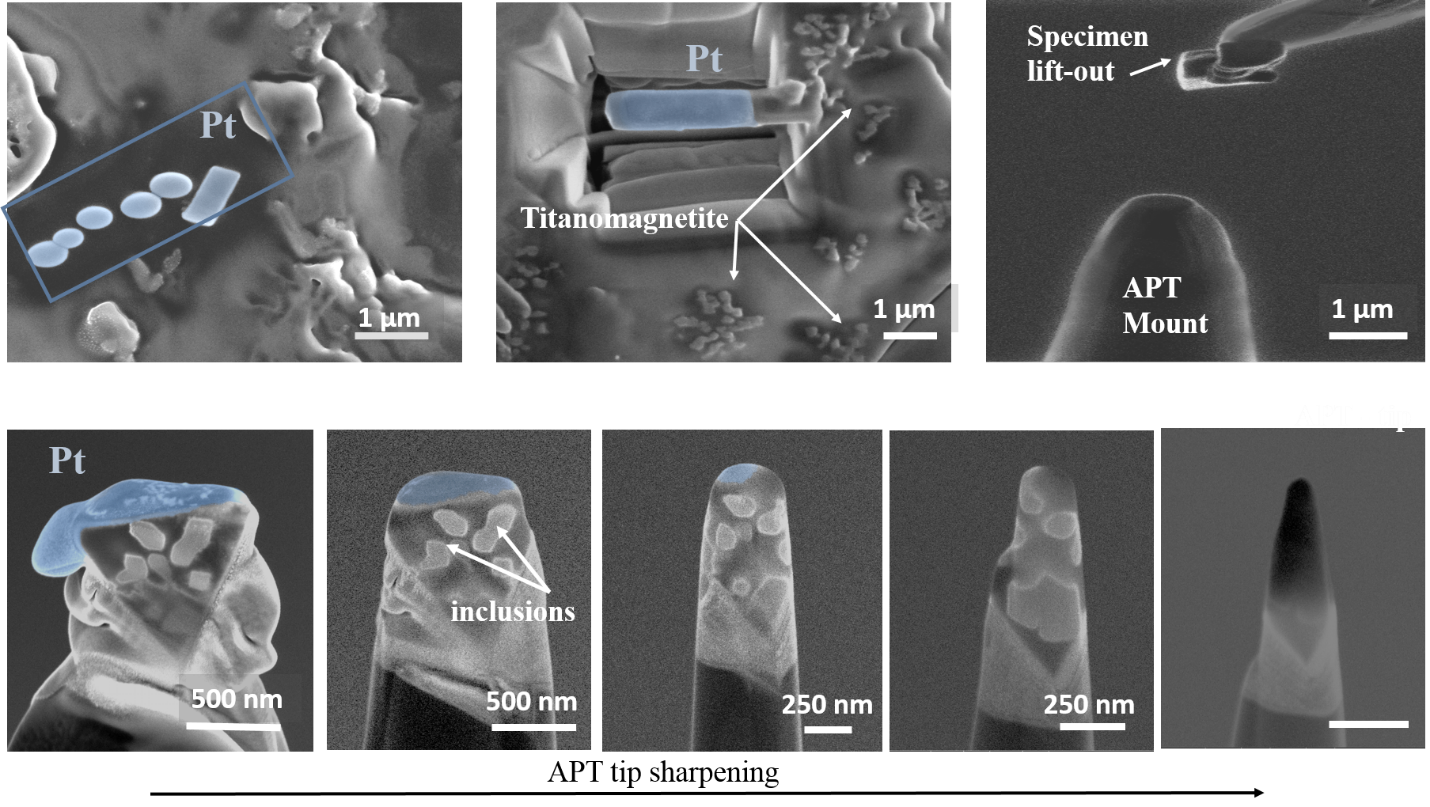


**Figure S13.** Sample preparation method for APT analysis including the steps taken to prepare an APT tip to investigate basaltic glass-titanomagnetite interfaces. The images show the location of the titanomagnetite minerals in relation to the basaltic glass matrix.

**Table S1.** Weight percent compositions of the basaltic glass matrix and magnetite inclusions as determined by EDX for Grains 1 and 2.

**Table S2.** Quantitative X-ray diffraction data obtained for three unreacted basalt control samples from the three-year weathering experiment.

**Table S3.** Weight percent compositions determined by APT within isolated volumes of a-c) Grain 1 and d-f) Grain 2. Elemental compositions are provided for the titanomagnetite phase within the green colored cylinder(s) whereas the grey colored cylinders represent the silicon-rich volcanic glass phase. The data sets were determined by background subtracted mass spectral analysis of mass peaks decomposed into elemental counts. The elemental compositions of the glass-phase are relatively consistent when comparing data within the isolated volcanic glass phase of the mixed glass-titanomagnetite data, and the data collected entirely within the volcanic glass phase.


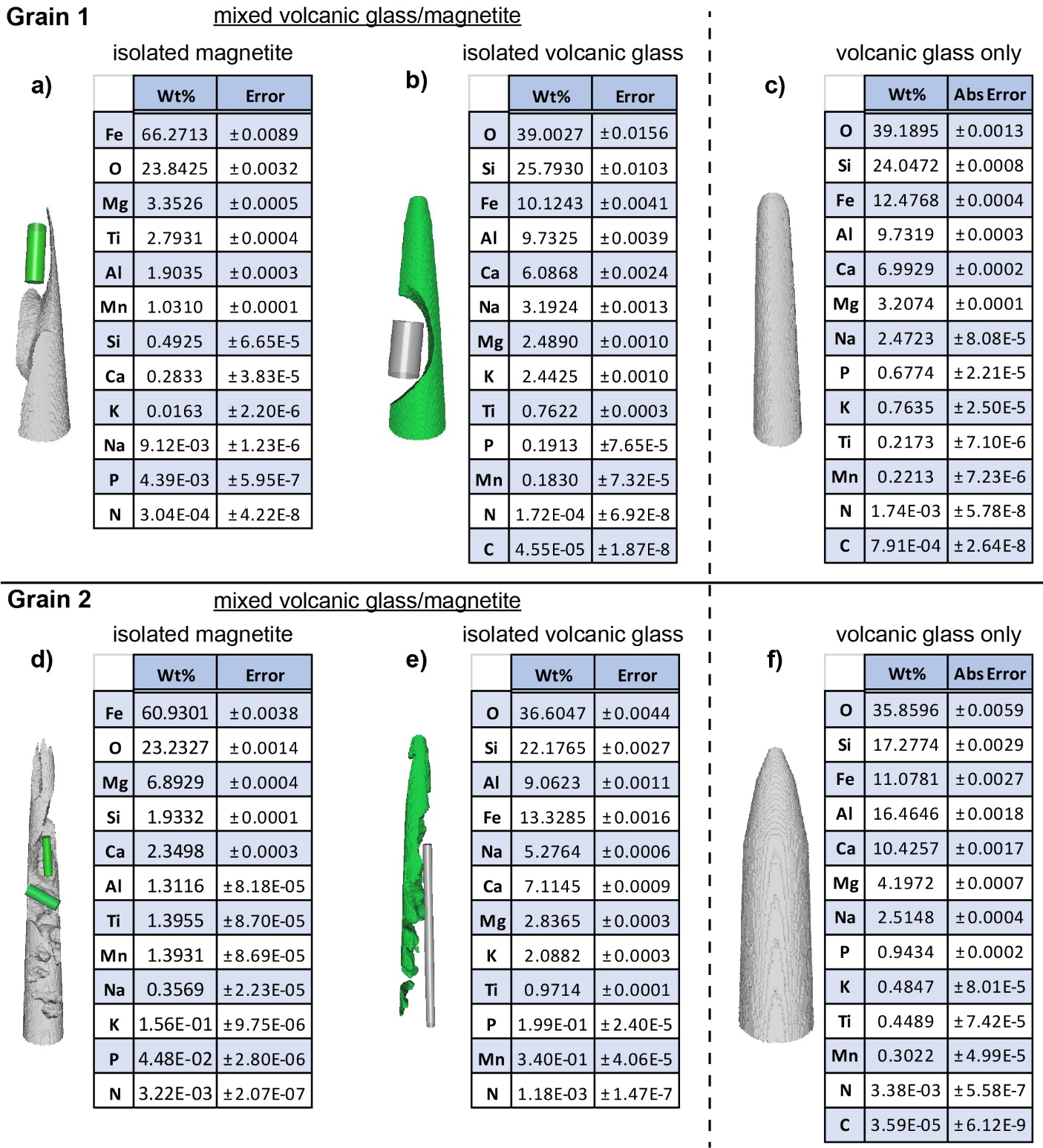


**Table S4.** Manual ion mass range assignments.
